# Excision-reintegration at a pneumococcal phase-variable restriction-modification locus drives within- and between-strain epigenetic differentiation and inhibits gene acquisition

**DOI:** 10.1093/nar/gky906

**Published:** 2018-10-13

**Authors:** Min Jung Kwun, Marco R Oggioni, Megan De Ste Croix, Stephen D Bentley, Nicholas J Croucher

**Affiliations:** 1 MRC Centre for Global Infectious Disease Analysis, Department of Infectious Disease Epidemiology, Imperial College London, London W2 1PG, UK; 2 Department of Genetics, University of Leicester, Leicester LE1 7RH, UK; 3 Parasites and Microbes, Wellcome Sanger Institute, Wellcome Genome Campus, Hinxton, Cambridge CB10 1SA, UK

## Abstract

Phase-variation of Type I restriction-modification systems can rapidly alter the sequence motifs they target, diversifying both the epigenetic patterns and endonuclease activity within clonally descended populations. Here, we characterize the *Streptococcus pneumoniae* SpnIV phase-variable Type I RMS, encoded by the translocating variable restriction (*tvr*) locus, to identify its target motifs, mechanism and regulation of phase variation, and effects on exchange of sequence through transformation. The specificity-determining *hsdS* genes were shuffled through a recombinase-mediated excision-reintegration mechanism involving circular intermediate molecules, guided by two types of direct repeat. The rate of rearrangements was limited by an attenuator and toxin-antitoxin system homologs that inhibited recombinase gene transcription. Target motifs for both the SpnIV, and multiple Type II, MTases were identified through methylation-sensitive sequencing of a panel of recombinase-null mutants. This demonstrated the species-wide diversity observed at the *tvr* locus can likely specify nine different methylation patterns. This will reduce sequence exchange in this diverse species, as the native form of the SpnIV RMS was demonstrated to inhibit the acquisition of genomic islands by transformation. Hence the *tvr* locus can drive variation in genome methylation both within and between strains, and limits the genomic plasticity of *S. pneumoniae*.

## INTRODUCTION

*Streptococcus pneumoniae* (the pneumococcus) is both a major bacterial respiratory pathogen (1) and an important model for studying the evolution of bacterial diversity in traits including antibiotic resistance, antigenic profile and virulence (2–4). This variation often represents the distribution of genomic islands (GIs), loci present in only a subset of the species (5–8). While many GIs are mobile genetic elements (MGEs), able to drive their own intercellular mobility, others are primarily exchanged through homologous recombination. This can be enabled by the transformation machinery of *S. pneumoniae*, which imports exogenous DNA in single-stranded form, thereby facilitating integration of new sequences that can introduce genes into the chromosome (9,10). The absence of CRISPR/Cas systems from pneumococci has been suggested as a further adaptation to promoting genomic plasticity (11). However, population analyses demonstrate non-prophage GIs are stably associated with lineages over decades (7). The low mobility of non-MGE GIs may partially reflect transformation being far more efficient at removing GIs than importing them (10,12). Also likely to be contributing to these evolutionary patterns are the diverse repertoire of restriction modification systems (RMSs) found in the pneumococcal population.

RMSs function by recognizing specific motifs, which are marked with modifications such as 6-methyladenine (^m6^A), 4-methylcytosine (^m4^C) or 5-methylcytosine (^m5^C) in the host genome by a methyltransferase (MTase). Double-stranded DNA lacking the endogenous pattern of modification, typically representing genetic material that has recently entered the cell, can be cleaved by the RMS endonuclease (REase) (13,14). Perhaps the best-known RMSs are Type II systems, composed of a cognate REase and MTase that both recognize the same motif, typically a 4–8 bp palindrome (13,15). This reflects their active forms being homodimeric and acting on double-stranded DNA. Although multiple Type II RMS can be found in the pneumococcal pangenome (7), the best-characterized examples are DpnI, DpnII and DpnIII (16). These alternative systems, encoded by genes found at the same genomic location in different isolates, all act at the motif GATC. The DpnI REase is unusual in targeting a modified motif, G^m6^ATC, which is generated by the MTase of the DpnII system; the DpnII REase has a complementary activity that cleaves GATC sites lacking this methylation (14,17). Similarly, the DpnIII REase cleaves motifs lacking the GAT^m5^C modification added by its cognate MTase (18).

Pneumococci also harbor a Type IV RMS, orthologous with the McrBC system of *Escherichia coli* that cleaves DNA if modified at low-specificity motifs (19). These genes have been implicated experimentally in protecting *S. pneumoniae* against phage infection (20). The other complete pneumococcal RMSs are Type I (21). These systems function as a holoenzyme usually composed of three subunit types: the HsdR REase, the HsdM MTase and the HsdS specificity subunit. HsdS directs the activity of both the MTase and REase toward a specific motif, the halves of which are separately bound by each of the specificity subunit's two target recognition domains (TRDs). Thus Type I RMSs recognize bipartite sequences in double-stranded DNA, methylated at two specific motifs separated by 6–8 non-specific bases (14,22). Studies of the Type I RMS EcoKI suggest the holoenzyme typically functions as an MTase when bound to a hemi-methylated motif, as arising following chromosomal replication; unmodified motifs are often cleaved by the REase subunits, although some Type I RMS MTases are just as active on such DNA (23–25).

RMSs can only block the transfer of sequence when they differ between the donor and recipient of horizontally transferred DNA. Hence they are most effective in this role when rare in a bacterial population, but less useful when common, meaning they are likely subject to negative frequency-dependent selection (8,26). Hence their ability to inhibit exchange of DNA is enhanced by phase variation, the ability to reversibly switch between phenotypes due to hypermutable genetic loci, which creates diversity in their specificity between otherwise isogenic, clonally related cells (14). For Type II RMSs, phase variation is typically limited to mechanisms that act as a reversible on-and-off switch (14), as coordinated alterations in the specificity of both the MTase and REase are difficult to engineer. An exception in *Helicobacter pylori* is a Type IIG RMS, as such consisting of a single polypeptide encoding both MTase and REase functions, able to switch between target motifs through frameshifting mutations (27). By contrast, Type I RMS are well-suited to phase variation that reversibly alters the motif they target, as changes to one, or both, TRDs of the HsdS protein simultaneously updates the specificity of both the MTase and REase (25). Multiple mechanisms of rearranging TRDs have been discovered in bacterial species, including ‘domain movement’ in *H. pylori* (28), in which recombination shuffles TRD-encoding sequences (TESs) between chromosomal loci, and ‘combinatorial variation’ in *Lactococcus lactis* (29), which can involve TESs on plasmids recombining with those encoded on the chromosome. In *S. pneumoniae*, a Type I RMS was identified as frequently undergoing rearrangements during culture (30), and was subsequently characterized as the inverting variable restriction (*ivr*) locus (7) encoding the SpnIII Type I RMS (31). The *ivr* locus is conserved in sequence across almost the entire species, containing five different TESs that can be combined into six different *hsdS* alleles through repeat sequence-mediated rearrangements, catalyzed by a site-specific tyrosine recombinase. Such phase-variable inverting Type I RMSs have been identified in taxa as diverse as *Mycoplasma pulmonis, Bacteroides fragilis* and *Listeria monocytogenes* (14).

A second pneumococcal Type I phase variable system, SpnIV, is encoded by the translocating variable restriction (*tvr*) locus, which was also identified using genomic data (7,31). Eight different TESs were identified within this locus across the pneumococcal population: four encoding N-terminal TRDs, labeled I-IV, and four encoding C-terminal TRDs, labeled i-iv. Unusually, all the Type I RMS coding sequences are found on the same strand, suggesting the rearrangements at this locus are not driven by the typical inversion-based mechanisms (14). This work characterizes the activities of this novel Type I phase variable system, and identifies the regulatory and recombination processes that drive its phase variation.

## MATERIALS AND METHODS

### *S. pneumoniae* strains, cultivation and transformation

All *S. pneumoniae* strains (Supplementary Table S1) were cultured at 35°C in brain heart infusion (BHI; Oxoid) liquid media supplemented with 0.32 μg ml^−1^ of bovine serum albumin (BSA, Sigma), or on solid agar media of the same composition, but supplemented with 200 U ml^−1^ catalase (Sigma). When measuring growth curves, 200 μl BHI was inoculated with 10^3^ colony-forming units and incubated at 35°C in a Biotek plate reader. Transformations were performed using 1 ml of the bacterial culture, collected at an OD_600_ of 0.10–0.15. Cells were incubated with 5 μl of 500 mM CaCl_2_ (Sigma), 500 ng of competence stimulating peptide one or 2500 ng of competence stimulating peptide two (Cambridge Bioscience Ltd) and 1 μg of genomic DNA at 35°C for 2 h. Samples were then spread on agar plates supplemented with antibiotics, as required: kanamycin 200 μg ml^−1^ (Sigma), rifampicin at 4 μg ml^−1^ (Sigma), erythromycin at 0.25 μg ml^−1^ (Sigma) or tetracycline at 2 μg ml^−1^ (Sigma). For the incubations with rifampicin and erythromycin, colonies were counted after 48 h of incubation at 35°C with 5% CO_2_. For selection using other antibiotics, 16–24 h of incubation was required. For measuring the ratio of Mega or Mega::*tetM* to rpoB* transformations, at least five replicate experiments were performed for each combination of donor DNA and recipient cells.

### Assaying *tvr* rearrangements by PCR

Ten milliliter BHI cultures were inoculated with individual *S. pneumoniae* colonies and grown overnight for 16 h at 35°C. Genomic DNA was extracted using Wizard genomic DNA extraction kits (Promega). For polymerase chain reactions (PCRs), 50–100 ng of genomic DNA was used as the template and added into Red-Taq reaction mix (Sigma) with hsdML or tvrAL as the forward primer, and a reverse primer specific to a particular TES (Supplementary Table S2). A touchdown PCR protocol was adopted to amplify all the possible bands with high specificity. During the first set of 15 cycles, the annealing temperature was reduced by 0.3°C per cycle from a starting temperature of between 57–59°C, depending on the sets of primers used, followed by 20 cycles using the expected annealing temperature. Elongation was conducted at 68–72°C for a period dependent on the expected amplicon sizes.

### Passage experiments

The *tvrT*::*tetM* mutants were grown from a single colony and then inoculated in liquid media supplemented with 2 μg ml^−1^ of tetracycline overnight. For the first passage round, 5% of the overnight culture was used to inoculate both fresh growth media without antibiotic, and growth media supplemented with tetracycline. After 16 h of growth in liquid media, cells were diluted and spread on agar plates prepared with and without tetracycline. Colonies within a 5 μl sample were counted on these different media, and the ratio of those carrying the *tetM* gene to the overall population number calculated. This was repeated to achieve 10 passages. The ratios of cells possessing *tetM* were monitored after the first, fifth, seventh and tenth passages. Assaying the length of the *tvr* locus in genomic DNA extracted from these cultures used primers hsdML and hsdRR in PCRs with the above conditions, but with a five minute extension time.

### RNA extraction and quantitative RT-PCR

Bacterial cells were harvested at an OD_600_ between 0.30 and 0.35. RNA extraction was performed as described previously (32). All RNA samples were further treated with amplication grade DNaseI (Invitrogen) according to the manufacturer's instructions. Reverse transcription of 1.5 μg of RNA was used to generate cDNA using the First-Strand III cDNA synthesis kit (Invitrogen). Samples were incubated at 25°C for 5 min, followed by annealing at 50°C for 30 min, further incubation at 55°C for 30 min and DNA synthesis at 70°C for 15 min. This was used as the template for qRT-PCR using the Powerup SYBR Green Master Mix (ThermoFisher) and QuantStudio 7 Flex System. Dilutions of genomic DNA were used to create standard curves to quantify the relative abundances of gene transcripts and validate the primers used in this RNA quantification assay. The *rpoA* gene was chosen as a reference to normalize data. Three technical replicates were performed on each of two biological replicates.

### Generation of DNA constructs

For removing the gene of interest, 0.6–1 kb PCR fragments of flanking regions of the gene were amplified and digested with the appropriate restriction enzymes (Promega) according to the manufacturer's instructions. The digested products were then ligated to the appropriate antibiotic resistance maker. Ligations of PCR products were performed overnight at 16°C with T4 DNA ligase (Invitrogen). Transformations either used the ligation mixture directly, or PCR amplicons generated using this solution as the template. All the details of the primers and the tested mutants for this study can be found in Supplementary Tables S1 and 2.

### Modification of the *tvrATR* locus

All the strains used in this study were transformed with a PCR product encoding a streptomycin-resistant allele of *rpsL*, denoted rpsL*. Transformants were selected on solid media supplemented with 100 μg ml^−1^ streptomycin. For those mutants constructed using the Janus cassette (33), successful transformants were first selected on 200 μg ml^−1^ kanamycin. Deletions were then selected using 100 μg ml^−1^ streptomycin, and tested for kanamycin sensitivity. Colonies were picked and PCR was used to check for the correct genotype. Attempts to remove the putative attenuator with the Janus cassette to assay its effects on transcription proved difficult, as its loss decreased the stability of the *tvr* locus, seemingly due to an increased rate of circular form excision. Therefore, modification of the terminator region involved first replacing *tvrT* and *tvrR* with the Janus cassette. The original *tvrT* and *tvrR* sequences were then re-inserted with the modified attenuator loci: a version lacking the hairpin (Δhairpin), a version that retained the original structure and appended four extra thymines to extend the polyuridine tract of the transcribed form (attenuator::T_4_), a version that lacked the putative TvrT toxin (Δ*tvrT* and Δ*tvrT* attenuator::T_4_), and the native sequence as a control (restoration). The *tvrATR* genes of each of these mutants were sequenced to ensure no unexpected changes had been introduced into the *tvr* locus.

### Assaying formation of circular molecules

Detection of circular molecules through PCR used primers Lcirc and Rcirc with the touchdown PCR protocol described above. For nuclease-based enrichment of the molecules, 10 ml BHI cultures of the R6x-derived mutants Δhairpin or attenuator::T_4_ were harvested at an OD_600_ of 0.4, and genomic DNA prepared. REase digestion of 25 μl genomic DNA used either ApaI (Promega) or HindIII (Promega) in manufacturer-specified buffers for 2 h at 37°C. Samples were then column purified using a PCR purification kit (Sigma) and digested with 30 μl φ29 exonuclease (New England Biolabs) for 30 min at 37°C according to manufacturer's instructions, prior to inactivation with 1 μl 25 mM ethylenediaminetetraacetic acid (Invitrogen) and heating to 70°C for 15 min. Quantification of *tvrR* and *rpoA* in the final samples used quantitative RT-PCR as described above, with three technical replicates performed on each of two biological replicates.

### Analysis of sequence motifs

Single molecule real-time (SMRT) sequencing (Pacific Biosciences) was performed as described previously (7), with the exception of *S. pneumoniae* RMV1 rpsL* *tvr*::*cat*, which was sequenced as part of a multiplexed library on the Sequel system (Pacific Biosciences). Reads were assembled using SMRTpipe 1.87.139483 within the SMRT Analysis 2.3.0 package. Reads were mapped against the appropriate assembly using pbalign 0.3.1 (https://github.com/PacificBiosciences); this was either the assembly from the same dataset, or the RMV1 rpsL*Δ*tvrR* assembly for the RMV1 rpsL* *tvr*::*cat* reads. Modifications were detected using ipdSummary version 2.3, and the motifs at which these occurred were identified with MotifMaker version 0.3.1. Accession codes for sequence data are reported in Supplementary Table S1, along with all modified motifs identified with a mean score above the threshold of 30 in Supplementary Table S3. The distribution of these motifs was analyzed using the sequence data from the Massachusetts collection of pneumococci (34). These clusters of orthologous sequences (COGs) have previously been categorized into the core genome, and those belonging to different classes of MGE: integrative and conjugative elements (ICEs), phage-related chromosomal islands (PRCIs) and prophage (7). Methylation motifs were identified within these sequences using Biopython (35) by parsing the individual coding sequences within each of the COGs, and counting the number of matches to regular expressions describing the specified target motif in both the forward, and reverse complement, forms. Density was calculated as the number of motifs across all coding sequences in the COG, divided by their total sequence length. Complementary analyses were run with R’MES (36), in which the full sets of sequences corresponding to each target motif definition were extracted with the gfam function; the distribution of each across the different COGs, divided according to the functional categories, was then quantified using the Gaussian and compound Poisson models. All data were plotted using ggplot2 (37).

## RESULTS

### A recombinase drives phase-variation of the *tvr* locus

**Figure 1.**
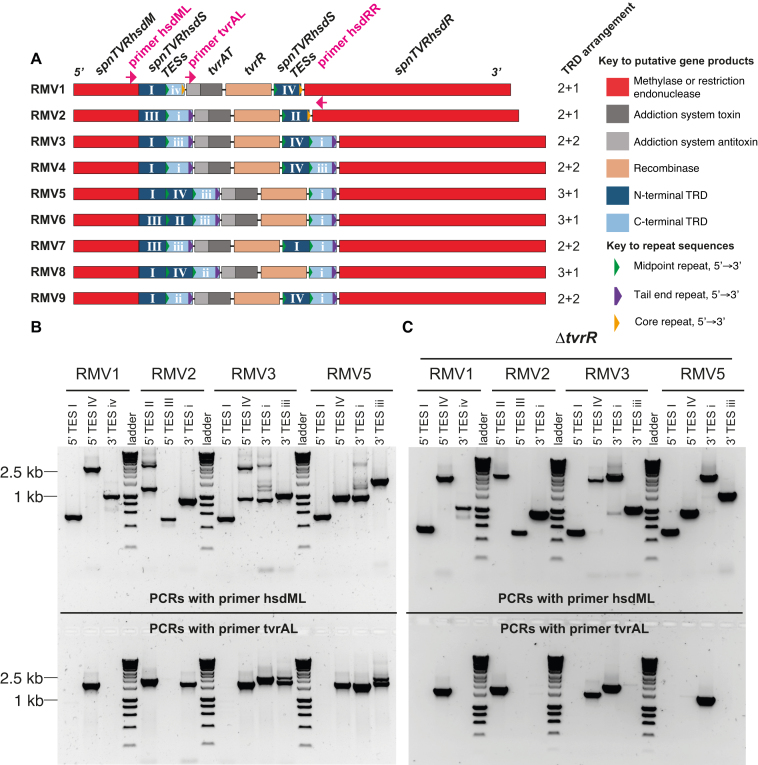
The *tvr* locus undergoes rearrangements driven by the TvrR recombinase. (**A**) Schematic of aligned *tvr* loci from isolates selected to represent the species-wide diversity of RMSs. These represent the 2+1, 3+1 and 2+2 arrangements annotated at the side. This shows the genes encoding the putative methylase (*hsdM*), endonuclease (*hsdR*), toxin-antitoxin system (*tvrAT*) and recombinase (*tvrR*). The TESs are shown in dark blue, if they encode N-terminal TRDs, or light blue, if they encode C-terminal TRDs. A functional *hsdS* gene is formed when two TESs of complementary types are joined into a single coding sequence. The two types of direct repeat in the locus are marked, with their consistent 5′ to 3′ orientation, along with the positions of two left primers and a fixed right primer, hsdRR (pink labels). (**B**) TES shuffling demonstrated by PCR amplicons generated using the fixed left primers in panel A and right primers specific for different TESs. The template for amplification was genomic DNA extracted from overnight cultures inoculated with a single colony. Extensive shuffling is evident in the *tvr* loci of the wild-type isolates, based on the presence of multiple bands and the presence of individual TESs shortly downstream of both fixed left primers. The exception is the TES immediately downstream of *hsdM*, which is not observed downstream of *tvrR*. (**C**) Reduced rate of shuffling in Δ*tvrR* mutants. Template DNA again originated from overnight cultures inoculated with a single colony. Much less variation is evident in the same backgrounds when *tvrR* was removed, based on the simpler banding patterns, reduced presence of TESs downstream of *tvrAT* and stronger high molecular weight bands, which no longer compete with shorter products in the PCR amplification.

Systematic sampling of *S. pneumoniae* from infant carriage has been conducted across multiple sites for vaccine surveillance purposes (38). Eleven isolates were selected to represent the diversity of pneumococcal RMSs identified using such population genomic data (38,39), with particular focus on the variation at the *tvr* locus (Figure 1A); these are referred to as ‘restriction modification variants (RMVs)’ one to eleven (Supplementary Table S1). At least one of these eleven isolates contained a TES for each of the four previously identified N-terminal TRDs (I–IV) and the four C-terminal TRDs (i–iv) at the *tvr* locus (sequences in Supplementary Table S4), enabling the identification of the SpnIV RMS’s target motifs. Found between the upstream *hsdM* MTase gene and downstream *hsdR* REase gene, the arrangement of the TESs could be defined relative to a central cluster of three genes: *tvrAT*, encoding a putative toxin-antitoxin system orthologous with *phd-doc* (Supplementary Figure S1), and *tvrR*, encoding a putative site-specific tyrosine recombinase. Three different arrangements were observed in the genome assemblies: two with two TESs upstream of *tvrATR*, forming a complete *hsdS* gene, with one downstream (denoted as ‘2+1’); three with ‘3+1’ arrangements, with the second and third TESs forming a complete *hsdS* gene upstream of *tvrATR*; and four with ‘2+2’ arrangements, with complete *hsdS* genes both upstream and downstream of *tvrATR*.

Within these *hsdS* genes, the TESs encoding the N- and C-terminal TRDs consistently occupied the 5′ and 3′ positions, in a manner likely directed by two types of repeat. The 5′ TESs were associated with an ∼18 bp ‘midpoint repeat (MPR)’ on one, or both, sides (Figure 1A). The 3′ TESs contained a longer ∼70 bp ‘tail end repeat (TER)’, encompassing the previously described orange repeats (7), in their downstream region. Both repeat types shared an ∼11 bp ‘core repeat’ (Supplementary Figure S1), which was present in RMV1 in the positions occupied by TERs in other isolates, as well as replacing one TER in RMV2 (Figure 1A). Based on this core section, all repeats shared the same orientation within the *tvr* locus, and have the potential to be targeted by the same site-specific recombinase, given that insertion sequence elements common in *S. pneumoniae* have inverted repeats between 12 and 29 bp in length (40). PCR assays were therefore used to test to what extent shuffling of TESs was affected by different arrangements and repeat types across these *tvr* loci. Left primers were fixed in the *hsdM* gene (hsdML), to detect TES rearrangements upstream of *tvrATR*, or *tvrA* gene (tvrAL), to detect TES rearrangements downstream of *tvrATR*. The right primers were designed to be specific to each of the TESs (Supplementary Table S2). The template genomic DNA was extracted from cultures grown for 16 h from a single colony of RMV1 or RMV2 (both 2+1), RMV3 (2+2) or RMV5 (3+1).

In RMV1, lacking full TERs, there was no evidence of TES shuffling: only single bands were generated for each right primer when combined with hsdML, and only TES IV occupied a position downstream of tvrAL (Figure 1B). In RMV2, with one full TER, two TESs appeared mobile. The hsdML and TES II primers generated a product greater than 2.5 kb, reflecting its expected position downstream of *tvrATR*, and a more efficiently generated product of around 1 kb, reflecting movement upstream of *tvrATR*. Analogously, TES i was detected in its expected position, near hsdML’s binding site, and downstream of *tvrATR*, based on the product generated with tvrAL. However TES III, adjacent to *hsdM*, showed no evidence of movement, which may reflect the absence of a repeat at the 5′ end. The TESs at the same position in RMV3 and RMV5 were also the only ones not to show evidence of shuffling with either hsdML or tvrAL. All the other TESs in these two isolates appeared on both sides of *tvrATR*, as reflected by the mixture of high (≥2.5 kb) and low (≤1 kb) molecular weight bands with hsdML for the TESs expected to be downstream of *tvrATR*, and the strong bands observed for all three of TES IV, i and iii for amplifications with tvrAL.

The role of the putative recombinase, TvrR, in driving these changes was tested by deleting the *tvrR* gene in these four isolates using the Janus cassette (33), a dual selectable marker that allows for genes to be replaced, and then the cassette itself to be removed. Genomic DNA was prepared as for the wild-type isolates, and the same PCRs run. Although very little difference was observed for RMV1, the pattern of PCR amplicons in the other RMVs indicated a greatly reduced rate of rearrangement. The clearest signal was that only TESs expected to be downstream of *tvrATR* generated strong bands with primer tvrAL, indicating a reduced shuffling of TESs relative to these central genes. Slower rearrangement rates were also indicated by the single bands observed with primer hsdML, particularly the higher molecular weight PCR amplicons that were no longer outcompeted by shorter alternatives (Figure 1C). However, limited phase variation in the positions of TRDs was still detectable in the Δ*tvrR* genotypes, as observed at the *ivr* locus following deletion of the analogous *ivrR* gene (31). Hence shuffling is greatly enhanced by the recombinase, but continues at a lower level in its absence.

### Identification of SpnIV and Type II RMS target motifs

Fourteen different *tvr* arrangements, ‘locked’ by deletion of *tvrR* where necessary, were characterized by SMRT sequencing. These confirmed the expected *tvr* genotypes and identified the methylated motifs throughout the chromosome. After accounting for the known targets of the SpnIII system (7,31), the remaining bipartite motifs modified to incorporate ^m6^A could be assigned to the *tvr* loci following two rules: first, that each TRD determined one half of the bipartite motif, as with other Type I RMSs; and second, that only the *hsdS* gene upstream of *tvrATR* was expressed in *tvr* loci with a 2+2 arrangement, with no evidence of the second downstream *hsdS* locus driving methylation. Furthermore, although the N-terminal TRDs II and IV had been separately classified in the original clustering of these sequences, owing to them being distinguished by four conserved amino acid substitutions (7), they actually recognized the same base pattern. These patterns within the SpnIV motifs were separated by 6–8 non-specific bases, depending on the combination of TRDs (Table 1 and Supplementary Table S3).

**Table 1.** Methylation motifs associated with each TRD of the SpnIV RMS

TRDs	Methylation motifs
N-terminal TRD I	GAY
N-terminal TRD II	TCA
N-terminal TRD III	TGA
N-terminal TRD IV	TCA
C-terminal TRD i	TATC
C-terminal TRD ii	RTAC
C-terminal TRD iii	TCC
C-terminal TRD iv	Non-functional due to the truncated C-terminal end

These are listed as they appear 5′ to 3′ in the bipartite motifs, with the sequence assigned to the N-terminal TRD at the 5′ end. The motifs are described according to the IUPAC code: ‘Y’ represents a pyrimidine, and ‘R’ represents a purine. The full set of motifs from which these were derived is shown in Supplementary Table S3.

The exception was an unusual approximately palindromic motif, CTBV ^m6^AG, identified in two independent mutants of RMV1: one Δ*tvrR*, and the other in which only part of *tvrR* had been removed (Supplementary Table S1). These both had *tvr* loci encoding an HsdS protein with TRDs I-iv. The most likely explanation seemed that the C-terminal TRD iv, which is similar to TRD ii but less than half its length, does not bind DNA. Instead, the N-terminal TRD I, found to recognize the motif GAY (Table 1), was able to bind a palindrome consisting of this motif in forward and reverse orientations, through acting as a dimer. The ability of an N-terminal TRD to functionally dimerize in place of an inactive C-terminal TRD has been experimentally demonstrated previously using EcoR1241 (41,42), which resulted in the recognition of a symmetrical, albeit still bipartite, motif. To test this, a RMV1 *tvr*::*cat* genotype was constructed and characterized through SMRT sequencing. This found the CTBV ^m6^AG to still be modified, suggesting a different MTase was responsible. Searches for a candidate enzyme present in RMV1, but absent from the other sequenced strains that lacked this particular modification, identified an orphan MTase (Supplementary Figure S2) similar to those in Type II RMSs, which would account for the palindromic nature of the motif. A search of REBASE found a similar, but truncated, MTase in *Streptococcus suis* (labeled M.Ssu478ORF7950P), predicted to target the motif CTRYAG (21). Hence the *tvr* locus of this isolate appears to be inactive, likely due to the short TRD iv rendering the HsdS protein non-functional.

The other non-bipartite methylation motifs could be attributed to Type II RMSs (Supplementary Table S3). The approximately palindromic sequences TCG^m6^AG, detected in RMV1 and RMV3, was assigned to SpnV (encoded by the sequences with accession code LK020705) (7); this is orthologous with the *S. pneumoniae* D39 SpnD39II system (31), found to recognize the same motif (21). The motif G^m6^ATGC, detected in RMV10 and RMV11, was attributed to SpnVI (encoded by the sequences with accession code LK020709) in Supplementary Table S3; this is orthologous with an RMS in REBASE predicted to target this motif, represented by the sequence M.Spn219ORF1273P. Similarly, the motif GG^m4^CCN_2-4_B was attributed to an RMS designated SpnVII (accession code: LK020710), only present in isolate RMV11. Although this motif is not precisely defined, this RMS REase matches the Pfam ‘RE_HaeIII’ domain (accession PF09556), which encompasses RMSs targeting motifs featuring GGCC sequences. A fourth candidate Type II RMS (encoded by the sequences with accession code LK020708) was also present in only one strain, RMV3, and therefore can be named SpnVIII, but could not be confidently assigned to a specific motif.

### The endonuclease activity of SpnIV inhibits transformation with genomic islands

**Figure 2.**
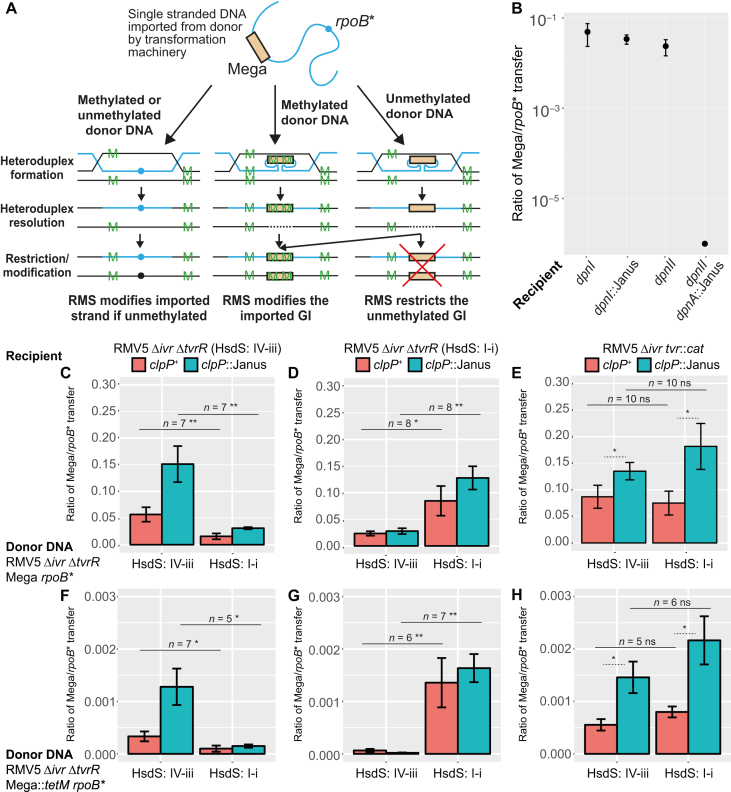
Inhibition of genomic island transfer by RMSs. (**A**) Design of the assay. Green ‘M’ characters represent sites at which DNA strands are modified. Donor DNA contains a Mega macrolide resistance cassette, and a rifampicin resistance SNP in *rpoB*. Import of the rifampicin resistance SNP results in a heteroduplex that is either fully or hemi-methylated, depending on whether the donor and recipient differ in RMSs targeting the recombinant locus. Neither often results in self-restriction, as hemi-methylated DNA is typically converted to a fully methylated duplex by an RMS MTase. By contrast, Mega import by transformation forms a single-stranded intermediate that, following second strand synthesis, is either hemi-methylated or unmethylated at RMS sites recognized by the recipient, depending on whether the donor and recipient differ in RMSs targeting the locus. An unmethylated duplex may also be methylated, but is also susceptible to self-restriction by an RMS REase. (**B**) To avoid such self-restriction, the DpnII system includes the single-stranded DNA methylase DpnA to ensure imported GIs are hemi-methylated rather than unmethylated. The plotted ratio was calculated from the number of macrolide and rifampicin-resistant transformants generated following transformation with donor DNA from R6x Δivr rpoB* Mega (expressing the DpnI RMS). The error bars show the standard error of the mean. Relative to the recipients with the same RMSs as the donor, no inhibition of GI acquisition was observed when *dpnI* was disrupted (recipient R6x *Δivr dpnI::*Janus) or or replaced with the intact *dpnII* locus (recipient R6x *Δivr dpnII*). However, disruption of *dpnA* within *dpnII* (recipient R6x *Δivr dpnII dpnA*::Janus) almost eliminated transformation with the GI. Panels (**C**–**E**) show transformation experiments in which donor DNA originated from RMV5 Δ*ivr* ΔtvrR rpoB* Mega cells, with a SpnIV specificity protein composed of TRDs IV-iii (recognizing three sites in Mega) or I-i (recognizing two sites in Mega). Recipient cells also had their *tvr* loci locked to express one of these specificity proteins. Each combination of donor and recipient cells is annotated with the number of replicates (*n*), and *p* calculated from a two-tailed Wilcoxon rank sum test: * (*P* < 0.05), ** (*P* < 0.01), *** (*P* < 0.001) and ns (non-significant). The error bars show the standard error of the mean. (C) Mega GIs from IV-iii donors were acquired more efficiently by IV-iii recipients, particularly if *clpP* was disrupted. (D) The complementary transformation shows Mega GIs from I-i donors were acquired more efficiently by I-i recipients. (E) Recipients without a functional SpnIV RMS acquired Mega GIs equally efficiently from donors with IV-iii and I-i SpnIV specificity proteins. The effects of *clpP* disruption were still evident, indicating they were independent of the SpnIV RMS. Panels (**F**–**H**) show the same experiments conducted with a construct generated by inserting a *tetM*-based DNA segment into Mega, ensuring the GI contained six recognition sites for the IV-iii and I-i SpnIV specificity proteins. This generally increased the inhibition of GI acquisition by transformation.

Having established the modification activity of the SpnIV system, a transformation-based assay was used to assay its ability to restrict imported DNA. Previous work (17) has identified a potential role for Type II RMSs in inhibiting the acquisition of genomic islands by transformation, despite the DNA being imported by the requisite machinery in single-stranded form, unless there are mechanisms in place to alleviate their activity when the bacteria are competent, as with the DpnII system. This is a consequence of RMSs tending to modify newly synthesized strands if they are part of a duplex hemimodified at the appropriate motifs, as after chromosomal replication (25,43). Hence the acquisition of resistance phenotypes caused by single nucleotide polymorphisms (SNPs) should not be affected by Type I RMSs (17,44), because only a single strand of the recipient replicon is displaced, meaning the recombinant sequence is at least hemi-modified. However, import of GIs on single-stranded DNA introduces novel sequence, which may be modified in a different pattern to endogenous DNA, if it originates from cell with a different set of RMS activities. If this is the case, when the complementary strand to the additional sequence is synthesized, it may not acquire the endogenous methylation pattern, rendering it susceptible to cleavage by RMSs; such chromosomal self-restriction would likely be fatal to the cell (Figure 2A).

An assay of the role of RMSs in inhibiting GI acquisition was therefore validated based on the previously observed effects of the DpnI and DpnII RMSs. This measured the relative rates of transfer of an ∼5.5 kb Mega GI, causing resistance to macrolides, and a SNP in the *rpoB* gene, causing rifampicin resistance. Both of these markers were integrated into the chromosome of the unencapsulated laboratory strain R6x Δ*ivr* (12), which was used as a donor of DNA in transformations of the parental R6x Δ*ivr* genotype, a mutant in which the DpnI system had been removed (R6x Δ*ivr dpnI*::Janus) and a mutant in which the DpnI system had been replaced with DpnII (R6x Δ*ivr dpnII*). As expected of large insertions, acquisition of the Mega GI was always less efficient than gain of the rifampicin resistance SNP (12), yet the ratio with which the two were acquired did not significantly differ between recipients. This demonstrates the intact versions of these systems do not impede gene acquisition by transformation compared with recipients that lack either (17). Yet following removal of the DpnII gene *dpnA*, encoding the single-stranded DNA methylase that modifies DNA from a DpnI donor to the same form as if it originated from a DpnII-carrying cell, recipient cells suffered a dramatic decrease in the efficiency with which they acquired the Mega cassette relative to the *rpoB* SNP. This likely represented the DpnII system cleaving one of the five target unmethylated GATC sites in the cassette following the post-integration synthesis of a complementary unmethylated strand (Figure 2B).

To test how the SpnIV system behaved in this assay, two otherwise isogenic Δ*tvrR* mutants were generated in the RMV5 background with *tvr* loci expected to express a functional HsdS protein containing TRDs I-i (recognizing the motif GAYN_6_TATC, which occurs in the Mega cassette twice) or IV-iii (recognizing the motif TCAN_7_TCC, which occurs in the Mega cassette three times). The Mega cassette and a rifampicin resistance SNP were introduced into both in order to assay rates of transfer. Complementary transformations were performed in which recipients, from which the potentially confounding *ivr* locus had been removed, received both markers from donors that were near-isogenic but differed in their methylation patterns. The relative rate at which the Mega GI was acquired was 3- to 6-fold higher when donor DNA had the same SpnIV-determined methylation pattern as the recipient compared to when they differed (Figure 2C and D). This difference was eliminated when the *tvr* locus was removed from the recipients (Figure 2E). This suggests most isolates that acquire the Mega cassette from a donor with a different *tvr* locus will be killed through cutting the newly integrated locus after it has been made double-stranded.

### Dependence of SpnIV activity on GI sequence and other cellular processes

To test whether this lower inhibition of gene acquisition relative to the *dpnII* Δ*dpnA* genotype might be indicative of the activity of a restriction alleviation mechanism, the *clpP* gene was also disrupted by insertion of the Janus cassette in these isogenic mutants (33). ClpP is a protease previously observed to regulate RMS activity through proteolysis (24,25) and known to be active during competence for transformation, which it downregulates (45). ClpP could therefore reduce SpnIV activity in competent bacteria, as well as facilitating switching between SpnIV specificities as part of phase variation, to avoid cutting the host genome. When the donor and recipient shared the same *tvr* arrangement, the *clpP*::Janus recipients acquired the Mega cassette at ∼2-fold higher rates relative to rifampicin resistance when compared to the equivalent *clpP*^+^ recipient cells (Figure 2C and D). Similar results were observed when the *tvr* locus was replaced by a chloramphenicol acetyltransferase in the recipients (RMV5 Δ*ivr clpP*::Janus *tvr*::*cat*), demonstrating the increased uptake of the Mega cassette relative to the rifampicin resistance SNP likely represented a change in the regulation of the transformation machinery, not a difference in the activity of the SpnIV system (Figure 2E). Consistent with this, the ratio of Mega cassette transfer to that of the SNP conferring rifampicin resistance was similar in *clpP*::Janus and *clpP*^+^ recipients when the donor and recipient cells had different *tvr* arrangements (Figure 2C and D). Hence, differing *tvr* arrangements between donor and recipients had a significantly bigger effect on the relative rate of Mega acquisition in *clpP*::Janus recipients relative to those in which *clpP* was intact. This implies the SpnIV system restricted the uptake of GIs in its wild-type form, an activity that was enhanced when acquisition of the Mega cassette was increased by removing the negative inhibitor of competence, ClpP. Hence SpnIV actively inhibits uptake of GIs by transformation.

Nevertheless, SpnIV presented a substantially weaker barrier to GI transfer than the *dpnA^−^* DpnII RMS. This observation could result from the lower frequency of the more specific sites targeted by the Type I RMS. A different GI was therefore engineered using a construct based on Mega, with an added *tetM* tetracycline resistance gene (∼2.5kb), thereby generating an ∼8 kb Mega::*tetM* composite GI containing six target motifs for both the IV-iii and I-i SpnIV specificity proteins. The greater length of this GI meant the absolute ratios of its acquisition relative to rifampicin-resistant transformants were lower than when assaying the transfer of the Mega cassette (12). Yet similar patterns of results were obtained as for the Mega GI alone (Figure 2 and Supplementary Figure S3), although the SpnIV-mediated inhibition of Mega::*tetM* GI transfer was detectably increased (46). For recipients expressing the IV-iii specificity protein, this rise was slight, whereas for those expressing the I-i specificity protein, inhibition of GI acquisition increased ∼10-fold. The inferred inhibition of transfer again disappeared when SpnIV was not expressed by the recipients. This implies the restriction activity of the SpnIV RMS increases with the number of target sites, although the per-site activity may vary between specificity proteins.

Experiments with the Mega::*tetM* construct in *clpP*^−^ recipients reproduced the observations that loss of this protease meant the ratio of GI to SNP transfer was elevated more when donor and recipient shared the same SpnIV RMS specificity, and that this increase in GI acquisition was independent of SpnIV, based on experiments with *tvr*::*cat* recipients. To test whether any of these results might by affected by DNA repair processes within the cell, the experiments were repeated in recipients lacking the mismatch repair gene *hexB*. This found the relative rates of exchange were not affected, with SpnIV still inhibiting GI movement (Supplementary Figure S3). However, the absolute values of the ratios were decreased, as the rifampicin resistance SNP was more frequently incorporated in the absence of mismatch repair. Therefore the SpnIV RMS inhibits the movement of GIs through transformation with a variable per-site restriction efficiency that appears substantially lower than that of the *dpnA*^−^ DpnII RMS.

### Shuffling occurs through repeat-mediated excision of a circular intermediate

**Figure 3.**
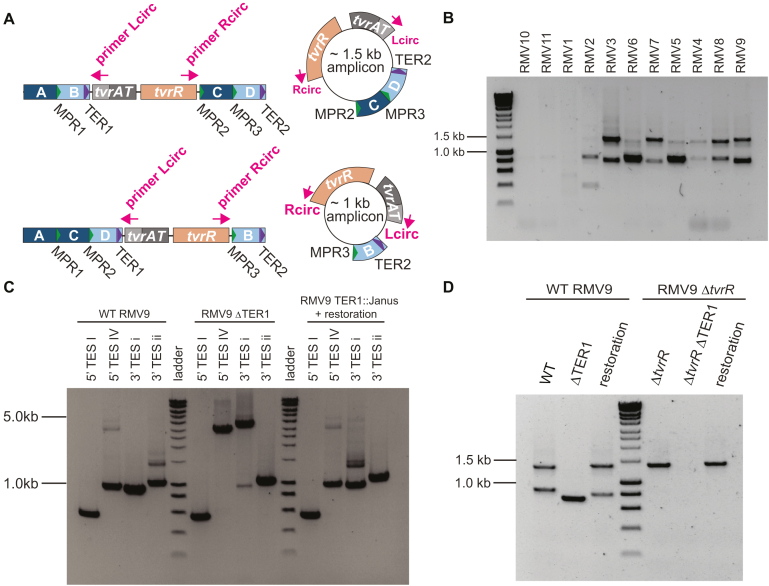
Characterization of the excision-reintegration shuffling mechanism occurring through repeat-mediated recombination. (**A**) Positioning of primers Lcirc and Rcirc, which are outward facing in the linear form of the *tvr* locus, and therefore are used to detect the putative circular intermediates. The smaller ∼1.0 kb product arises when only one TES lies between TER1 and TER2, through which the excision is expected to be possible. When two TESs lie between the repeats, the larger ∼1.5 kb circular intermediate is generated. (**B**) Gel showing PCR amplicons generated by the outward-facing primers, indicating the presence of the predicted circular intermediate forms of the excision-reintegration mechanism. In the three strains lacking TER1 or TER2 (RMV1, RMV10 and RMV11), no product is detected. Atypical results for RMV2 are likely to be a consequence of its TER2 being truncated to just the core repeat (Figure 1A). (**C**) Role of TER1 in facilitating *tvr* shuffling. Rearrangements were detected using PCR amplicons generated using the fixed left primers in Figure 1A and right primers specific for different TESs. Rearrangements of TESs were greatly inhibited following the deletion of TER1 (RMV9 ΔTER1). Shuffling activity returned once the repeat had been restored. (**D**) Role of TER1 in facilitating excision of the circular forms of the *tvr* locus. Using the outward facing primers, only a single size of circular form was detected in RMV9 ΔTER1. This likely reflects excision via the MPRs. In a Δ*tvrR* background, no circular forms were detected in the ΔTER1 mutant, indicting efficient excision through the MPRs requires TvrR.

Hence, within an originally isogenic cell population, shuffling at the *tvr* locus will generate barriers to exchange of GIs by transformation. These rearrangements seem likely to be primarily driven by the putative site-specific tyrosine recombinase TvrR and the two types of repeat sequences, based on the reduced rearrangements observed in the Δ*tvrR* genotypes and the wild-type RMV1, the *tvr* locus of which lacked TERs (Figure 1B). By contrast, RMV9 exhibited extensive shuffling (Figure 3C); however, when the upstream TER was deleted (RMV9 ΔTER1; Figure 3C) or disrupted (RMV9 TER1::Janus; Supplementary Figure S4), much lower levels of variation were evident, despite sequencing demonstrating the *tvrR* gene was unmodified. Restoration of the original TER sequence reinstated shuffling behavior, consistent with the changes at the repeat alone being responsible for the locking of the locus (Figure 3C).

The inhibition of *tvr* shuffling in the absence of two direct repeats suggested these motifs may facilitate excision of the central part of the *tvr* locus in a circular form, analogous to the excision of prophage through *att* sequences. To test this, the RMV isolates were tested with the outward-facing primers Rcirc and Lcirc, which should only produce an amplicon if this region has a circular topology (Figure 3A). As expected, all isolates encoding four TRDs within the *tvr* locus produced two amplicons: one of ∼1.0 kb, corresponding to the excision of *tvrATR* and a single TRD from a 3+1 configuration; and one of ∼1.5 kb, corresponding to the excision of *tvrATR* and two TRDs from a 2+2 configuration (Figure 3B). Sequencing of these amplicons confirmed the expected presence of *tvrR* and alternative TESs. By contrast, no circular forms were detected in isolates RMV1, RMV10 or RMV11, all of which lack full-length TERs. Similarly, only a weak band was evident for RMV2, in which one TER was reduced to just the core repeat sequence (Figure 1A), and the RMV9 ΔTER1 mutant (Figure 3D). This suggests circularization can also occur through the MPRs, particularly when three are present in 2+2 or 3+1 arrangements, as in RMV9.

TvrR was hypothesized to be involved in the excision and reintegration of these circular forms, based on its role in driving *tvr* rearrangements. RT-PCR confirmed the *tvrR* gene was transcribed whether in a linear or circular topology (Supplementary Figure S5), indicating it had the potential to be active in both processes. Yet testing for excision in the locked Δ*tvrR* mutants found circular forms were still detectable, albeit at a reduced level (Figure 3D). This suggested TvrR was likely involved in excision of the circular forms, but that other recombinases may also mediate the same processes. To test this, the locked *tvr* locus of RMV4 was introduced into the highly transformable laboratory strain R6x Δ*ivr* (12), and a series of double mutants generated to screen for absence of circular intermediates. Tested genes included single and double mutants of the replication recombinases *xerS* (spr1056) and *xerD* (spr1692), and the homologous recombination-related genes *recA, dprA, recG, rexAB* and *recU*. However, all of these mutants still produced detectable levels of the circular intermediates (Supplementary Figure S6). That this PCR amplicon was generated in a *recA*^−^ background rules out any intragenomic homologous recombinations, such as unequal crossing-over, as an explanation for the detection of a product with these outward-facing primers. Hence an as-yet uncharacterized recombination pathway appears able to inefficiently cause excision in the absence of TvrR.

The only double mutant in which circular forms were no longer detectable was RMV9 ΔTER1 Δ*tvrR* (Figure 3D), suggesting TvrR-independent excision can only occur efficiently at the longer TERs, whereas TvrR-dependent excision may also occur at the shorter MPRs. Correspondingly, very little evidence of continued shuffling at the *tvr* locus could be identified in this double mutant (Supplementary Figure S4). Therefore, as at the *ivr* locus (31), it appears the recombinase is necessary to drive rearrangements involving the short repeats, but an uncharacterized recombination pathway appears able to inefficiently cause recombination between the long tandem repeats.

### TvrAT proteins suppress shuffling

**Figure 4.**
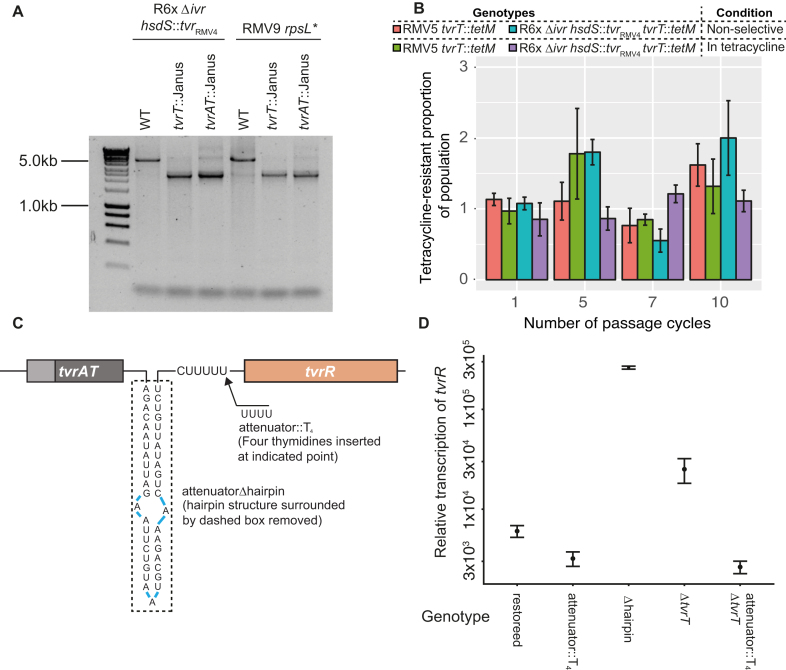
Mechanisms regulating the *tvrR* gene. (**A**) PCR amplicons spanning the linear *tvr* locus generated using primers hsdML and hsdRR following overnight growth initiated from a single colony. These amplicons are expected to be ∼5 kb when the locus is intact, or ∼2.5 kb following excision of a circular molecule including *tvrATR* and TESs. Replacement of *tvrAT* or *tvrT* alone in two different backgrounds increased the prominence of the smaller band relative to the native version of the locus, indicating the circular forms were either being excised more rapidly, or reintegrated less often, potentially due to loss through segregation. (**B**) Stability of the *tvr* loci in which *tvrT* was replaced by the *tetM* tetracycline resistance gene. The proportion of cells exhibiting tetracycline resistance at four different stages of a passage are shown in two different strains. These results show there is no evidence for loss of the *tetM* genes throughout the passage experiment, regardless of whether the cells were grown in selective or non-selective media. This indicates the TvrAT system is not necessary to maintain the stability of the *tvr* locus. (**C**) Structure of the Rho-independent terminator-like structure between *tvrAT* and *tvrR* suggested to act as a transcriptional attenuator. The hairpin structure, removed in the mutant Δhairpin, and the polythymidine tract, into which extra thymidines were inserted in the attenuator::T_4_ mutant, are indicated. (**D**) Effect of putative attenuator modifications on *tvrR* transcription. Transcription was measured through qRT-PCR. Removal of the hairpin significantly increased *tvrR* expression, demonstrating biologically relevant transcriptional attenuation. Removal of *tvrT* also elevated expression, demonstrating it acted to repress *tvrR* transcription. Elongating the polythymidine tract to terminate transcription reduced *tvrR* expression in both the wild-type and Δ*tvrT* backgrounds.

The excision of a circular intermediate raises the possibility that this separate DNA molecule could be spontaneously lost, resulting in fixation of the shortened form of the locus. Yet the full *tvr* locus is stable over long evolutionary timescales (7). This suggests a potential function for the *tvrAT* proteins, which are orthologous with the Doc-Phd toxin-antitoxin system (Supplementary Figure S1). Doc blocks translational elongation unless its labile partner Phd-type antitoxin is present; therefore post-segregational loss of the encoding pair of genes results in the stable toxin persisting after the antitoxin is degraded, killing the host cell (47). Such addiction systems stabilize genetic loci, including slowing the rate of plasmid curing (48) and therefore the putative *tvrAT* toxin-antitoxin pair seemed a likely mechanism by which circular forms carrying these genes might be retained, maintaining the intact *tvr* locus. To test for evidence of this function, *tvrAT* was replaced with a Janus cassette in RMV9, in which TRDs were detectably shuffled at high frequency, and in an R6x Δ*ivr* strain carrying the *tvr* locus of RMV4 (R6x Δ*ivr hsdS*::*tvr*_RMV4_). In the wild-type *tvrAT*^+^ cultures, an ∼5 kb amplicon was generated using primers binding in *hsdM* and *hsdR*, corresponding to the full-length *tvr* locus (Figure 4A). However, in the *tvrAT*::Janus mutants, a shorter amplicon around 2.5 kb became more prominent following overnight growth. Sequencing of this amplicon revealed that this detected *tvr* locus only carried *hsdM, hsdR* and varying sequences for *hsdS* genes, consistent with loss of the circular forms. Similar results were obtained following disruption of *tvrT* only with a Janus cassette in both backgrounds (Figure 4A). This phenotype was observed independently of whether *tvrT* was disrupted by a Janus cassette, or the gene was deleted, indicating these observations were not a consequence of changing the spacing between repeat sequences (Supplementary Figure S7). Concurring with this observation, neither disrupting *tvrR* with a Janus cassette, nor deleting *tvrR*, had the same effect (Supplementary Figure S7). We therefore hypothesized that circular intermediate forms were rapidly lost, resulting in a shortened *tvr* locus, unless an addiction system stabilized the plasmid-like structure (47).

The rate at which this shortened *tvr* form arose was quantified by replacing *tvrT* with the *tetM* resistance marker in RMV9 and R6x Δ*ivr hsdS*::*tvr*_RMV4_ (Figure 4B). Resistance to tetracycline mediated by TetM is specific to the cell carrying the gene, without causing inactivation of the antibiotic throughout the media, making it appropriate for quantifying the prevalence of resistance in mixed populations. Assuming progressive, irreversible loss of the circular forms during culture, it was expected that these extrachromosomal DNA molecules would fall in prevalence more quickly in non-selective media in the absence of the addiction system toxin, measurable through a decline in the frequency of tetracycline-resistant cells in liquid cultures. However, in the presence of tetracycline, the resistance marker should be essential, resulting in the retention of the full-length *tvr* locus, including *tetM*. Therefore, passage experiments were conducted over ten days in selective and non-selective liquid media. However, the proportion of tetracycline-resistant cells did not differ between the two conditions (Figure 4B). Furthermore, PCR amplicons indicating the presence of circular forms were still detected in the mutants collected after 5 or 10 days of the passage (Supplementary Figure S8). Hence this experiment did not find evidence of TvrAT functioning as a toxin-antitoxin system.

To further test for evidence of TvrAT acting as an addiction system, growth curves were measured for RMV5 genotypes with disruptions of *tvrT, tvrA*, or *tvrAT* (Supplementary Figure S9). The loss of TvrA did not result in substantially reduced logarithmic growth rates, as might be expected for an addiction system, although there was a faster reduction in cell density during stationary phase, relative to the wild-type background. Although loss of TvrT appeared to slightly elevate fitness *in vitro*, this was not consistent with deletion of *tvrAT*, which behaved very similarly to the wild-type bacterium. Additionally, the *tvrAT* genes were efficiently deleted through recombination (Supplementary Figure S9). This was tested by transforming RMV5, RMV5 *tvrAT*::Janus and RMV5 *spnTVRhsdR*::Janus with DNA containing *tvr*::*cat* and *rpoB* markers. The ratio of chloramphenicol-resistant to rifampicin-resistant transformants therefore measured the efficiency of *tvr* locus deletion, which was found to be similar, regardless of whether *tvrAT* was present. By contrast, disrupting *spnTVRhsdR* increased the efficiency of *tvr* deletion, which may reflect a SpnIV target motif within the *cat* gene causing self-restriction of wild-type or *tvrAT*^−^ cells that imported this resistance marker. Therefore, while it cannot be ruled out that the TvrAT proteins function as an addiction system during some phases of growth, the effects observed in these experiments suggest the main role of these proteins is suppressing the excision of the central section of the *tvr* locus.

### Regulation of *tvrR* expression by RNA and proteins

Sequence analyses suggested *tvrATR* were transcribed as an operon from a single promoter (Figure 4C) with a potential transcriptional attenuator separating *tvrAT* from the downstream *tvrR*. RNAfold (49) predicted this attenuator would form a hairpin of 19 paired bases, followed by five uridines; hence when transcribed it would resemble a weak Rho-independent terminator, which typically has a stem of 7–20 bp, followed by a 7–9 nt long polyuridine tract (50). A series of mutants were constructed in the R6x Δ*ivr* background carrying the *tvr* locus of isolate RMV4 to test the role of this RNA structure in relation to *tvrATR*. Mutants were generated that lacked the hairpin (Δhairpin), appended four extra bases to extend the polyuridine tract (attenuator::T_4_), or restored the original sequence (restoration); these changes were all verified by sequencing. The rate of excision relative to a Δ*tvrT* mutant was assayed by amplifying the *tvr* locus as previously (Supplementary Figure S10). This found both Δhairpin and Δ*tvrT* were associated with elevated levels of the shorter locus, presumably reflecting more rapid excision of circular forms (Supplementary Figure S5). This is consistent with TvrT and the hairpin limiting expression of TvrR, which catalyses the excision. To test whether the Δ*tvrT* mutation had effects independent from the attenuator structure, the attenuator::T_4_ Δ*tvrT* double mutant was constructed. No increase in the shorter form of the *tvr* locus was detectable, suggesting TvrT acted to suppress transcription of *tvrR*, and its loss could be complemented by strengthening the attenuator's ability to terminate transcription. Disrupting *tvrA* also did not detectably increase the prevalence of the shorter *tvr* locus, indicating TvrA was not necessary for TvrT’s function (Supplementary Figure S11). Consistent with TvrA being less important in regulating the locus, the *tvrAT*::Janus mutant behaved similarly to that lacking *tvrT*.

Quantitative RT-PCR was used to confirm these changes represented alterations in levels of *tvrR* expression (Figure 4D and Supplementary Figure S12). These experiments found *tvrR* transcription was elevated by more than 40- and 4-fold in the Δhairpin and Δ*tvrT* mutants, respectively, relative to the strain with the restored locus, carrying the native *tvrATR* and attenuator site. Conversely, both attenuator::T_4_ and attenuator::T_4_ Δ*tvrT* mutants exhibited almost 2-fold lower *tvrR* transcription, demonstrating that extension of the polythymidine tract to lengths associated with Rho-independent terminators reduced the transcription of *tvrR* relative to the native locus. Consistent with the amplification of different lengths of the chromosomal *tvr* locus in these mutants, increased *tvrR* transcription was found to correlate with elevated levels of circular form excision (Supplementary Figures S13 and 14). Genomic DNA from both the Δhairpin and attenuator::T_4_ mutants was extracted and sequentially digested with ApaI, which does not cut within the *tvr* locus of either genotype, and φ29 exonuclease, which efficiently digests linear DNA. Quantitative RT-PCR of the processed samples found the concentration of *tvrR* DNA to be over 4-fold higher in the Δhairpin, but not the attenuator::T_4_, mutant. This is consistent with *tvrR* sequences being enriched on circular molecules, resistant to exonuclease digestion, when the attenuator structure was disrupted. As a control, no such difference in the concentration of *tvrR* DNA was observed when HindIII, predicted to cut the circular intermediate, was used in place of ApaI. Hence the rate of *tvr* shuffling through excision-reintegration appears to be suppressed by both protein and RNA structures in pneumococci.

## DISCUSSION

**Figure 5.**
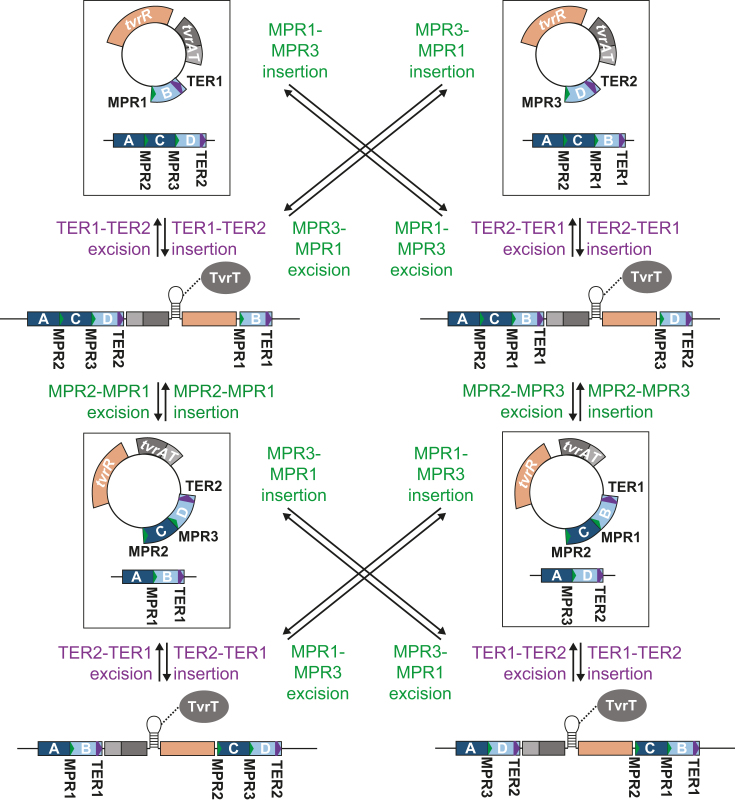
Proposed mechanism of excision-reintegration phase variation. In the linear form of the *tvr* locus, *tvrR* expression is repressed by the transcriptional attenuator and TvrT. Although this interaction is represented as being direct here, there are alternative possible mechanisms. From the linear locus, TvrR-mediated excision of a circular form can occur via the MPRs or TERs; a TvrR-independent mechanism can also operate on the longer TERs. If TvrR-mediated site-specific recombination integrates the circular molecule at the same repeat from which its was excised, then the original locus arrangement is restored. However, integration at a different repeat shuffles the TRD-encoding sequences. Hence a typical *tvr* locus with four TESs has four possible arrangements, with the TES immediately downstream of *hsdM* unmoved in each.

This work expands the characterized repertoire of pneumococcal RMSs beyond the Dpn and SpnIII systems, which respectively target GATC and one of a fixed set of six sequences in almost all pneumococci, to the GI-encoded Type II RMS and Type I SpnIV RMSs, which exhibit greater population-wide diversity. The motifs recognized by the SpnIV system were somewhat limited compared to the previously hypothesized range of specificities (7): two of the N-terminal TRDs suspected of being functionally distinct actually recognized the same motif; the C-terminal TRD iv was not found to facilitate targeting of particular sequences, and in loci with a 2+2 arrangement, only the upstream *hsdS* gene was active. Such an interpretation is consistent with the previously observed methylation motifs in isolates CH2060 (GATAN_6_RTC), ND6010 (GGAN_7_TGA) (7) and WCH16 (GAYN_6_TATC) (14), which can be explained through expression of the upstream *hsdS* gene, based on the TRD specificities described here. Therefore for any given isolate, the SpnIV RMS can shuffle between fewer functional states than the six observed at the SpnIII locus: *tvr* loci with four TESs can alternate between four different specificities, and those with three TESs can switch between two different specificities (Figure 5). However, the variation in TES content between *tvr* loci means there are at least nine possible specificities observed across the species, assuming the three different N-terminal and C-terminal TRD specificities identified here are all compatible with one another. Unlike the more conserved SpnIII RMS, there are many isolates that lack a functional version, such as D39, which represents further variation across the population. These non-functional versions could stably co-exist with the diversity of active systems in a single population if the corresponding restriction phenotypes be subject to negative frequency-dependent selection, under which scenario each *tvr* allele is most advantageous to its host cell when it is rarest (26,51). Hence this system has the potential to have an extensive impact on epigenetic variation and horizontal DNA transfer between isolates.

Despite this, there was little evidence of SpnIV-driven restriction avoidance (51): the depletion of RMS target motifs from the genome (Supplementary Figure S15). Analyses of motif distribution were run on the core genome, and the three types of MGE common in pneumococci (7): ICE, including the large transposons that commonly carry antimicrobial resistance; prophage; and PRCIs, non-autonomously mobile viral elements. When compared to the distribution of control motifs of the same base composition, there was little evidence of restriction avoidance in either the core genome or MGEs, apart from a slight signal in PRCIs. Motif sequence frequency analysis with R’MES (36) concurred that there were few signs of avoidance across these functional categories. Although the GATC sequence targeted by the three Dpn RMSs had a similar distribution to a control motif (Supplementary Figure S15), the R’MES analysis found it to be significantly depleted across both MGEs and, more strongly, the core genome (Supplementary Figure S16 and 17). However, in absolute terms, the short GATC motif was still far more frequent, occurring over 3500 times in each of the genome assemblies in this study, while the SpnIV motifs were detected between 595 and 1612 times in the respective RMVs (Supplementary Table S3).

In the absence of protective methylation, the DpnII REase appears to target these more common sites more efficiently than the SpnIV RMS cleaves its recognition motifs. Testing the nuclease activity of SpnIV and DpnII required assaying these RMSs' roles in inhibiting acquisition of genes by transformation, owing to the difficulties in experimentally assaying MGE infection in pneumococci. This work reproduced the findings of an elegant previous study of how the DpnI and DpnII RMSs facilitate exchange of GIs via the transformation machinery, while still having the potential to block movement of phage (17). This was suggested to provide a mechanism by which pneumococcal RMSs could avoid inhibiting advantageous acquisitions of genes, allowing cells to potentially adapt to new environments, as expected in an individual-centric evolutionary interpretation. Equally, the same mechanisms can be interpreted in a gene-centric evolutionary framework, as they prevent the RMS from killing cells in which it is present through self-restriction, should they acquire a GI by transformation, advantageous or not. The *tvr* locus does not obviously include a similar mechanism for restriction alleviation, a decrease in restriction activity against invading DNA (52), during transformation, When considering optimal evolutionary strategies, this neither seems consistent with the set of individual-centric interpretations in which new loci are acquired to facilitate adaptation to niches, nor the gene-centric perspective of preventing host cell death owing to incidentally acquired novel loci. One advantage from the latter perspective is the apparently selfish behavior, sometimes associated with RMSs (51), whereby the SpnIV RMS seems to cause self-restriction at target sites in DNA that replaces an active *tvr* locus, but this likely represents a rare circumstance. Furthermore, this self-restriction was observed to be relatively inefficient, such that replacement of the *tvr* locus was inhibited only around 2-fold (Supplementary Figure S9). Additionally, population genomic data demonstrate the SpnIV RMS can be inactivated *in vivo* without causing cell death, with different ICEs inserting into the *tvr* locus *hsdM* gene in *S. pneumoniae* AP200 and an isolate from the Maela pneumococcal collection (Supplementary Figure S18) (53,54). Such routine disruption of the *tvr* locus *in vitro* and *in vivo* is in contrast to the addiction system-like behavior observed for some Type II RMSs (47). Given this comparative inefficiency of SpnIV-mediated self-restriction, an alleviation mechanism may be of only limited benefit to the cell. This may be compounded by the difficulty of responding to the rapidly changing specificity of SpnIV.

Hence the simplest explanation for the apparent absence of a specific alleviation system may be the weaker selection pressure relative to that driving the evolution of DpnA in the DpnII RMS. Most insertions imported from other strains by transformation are short (9,12) and occur on a timescale of years or decades (9). While these recombinations may often feature the common GATC motif targeted by the DpnI and DpnII RMSs, they would be unlikely to include one of the rarer SpnIV motifs, meaning the cost of self-restriction is rarely experienced. However, the target sites would still be expected to feature on MGEs the size of a full-length phage (7), which infect *S. pneumoniae* on the timescales of weeks or months (10), meaning the SpnIV RMS may often be advantageous. In addition, the SpnIV activity against imported GIs is clearly weak relative to the DpnII *dpnA*^−^ locus. This may reflect an intrinsic property of SpnIV: RMSs have been suggested to vary in their restriction efficiency, the probability that a target site will be cleaved rather than methylated (26,46). SpnIV likely has low restriction efficiency, reflecting the high modification activity needed to avoid self-restriction during phase variation. By contrast, DpnII appears to have higher restriction efficiency, consistent with the evolutionary stability of this locus (7) meaning unmodified DNA is highly likely to represent imported exogenous DNA, rather than a recent change in RMS specificity.

Despite the lower activity per imported DNA molecule, SpnIV is still likely to have a substantial effect on the population-wide distribution of loci. Assuming *S. pneumoniae* cells are typically part of a clonally related population (10), the greater diversity of RMS specificities that can accumulate over short timescales through phase variation means SpnIV can act to block the intercellular spread of GIs, such as lysogenic phage. The conservation of Dpn RMSs over much longer evolutionary timescales means they cannot prevent such within-strain movements (7). However, the *tvr* locus’ rate of variation appears to be under selection to be lower than the maximum possible rate, given its regulation by transcriptional attenuation and proteins orthologous to the toxin-antitoxin system Doc-Phd. Although we have no evidence of a direct nucleic acid–protein interaction, the archetypal *doc-phd* locus was autoregulated by the proteins recognizing a palindromic operator sequence that blocked transcription initiation (55), suggesting TvrAT could directly bind the attenuator locus. The TIGRFAM associated with the Phd homolog, TIGR02609, is noted as being enriched on chromosomes rather than plasmids, suggesting transcriptional regulation could be its primary role, consistent with our data (56). There is an additional intrinsic limitation on the variation rate imposed by the excision-reintegration mechanism proposed in Figure 5. While a single exchange between repeats at the *ivr* locus will always cause a change in specificity, two recombinations are required to both excise and reintegrate the circular intermediate at the *tvr* locus; furthermore, this integration-excision process can regenerate the original arrangement of the locus.

Ultimately, however, the variation of this locus may be constrained by the epigenetic consequences of DNA modification on gene expression. In the absence of any horizontal DNA transfer, particular arrangements of *tvr* loci dominated in independent cultures of different strains, making isolation of rarer alleles difficult. A notably high level of rearrangement was apparent in RMV9, which lacked a functional MTase. These observations are consistent with the shuffling of *hsdS* genes being constrained by epigenetic consequences for transcriptional patterns within the cell, which are alleviated in the absence of a functional holoenzyme. Hence the *tvr* locus variation in a natural clonally related pneumococcal population is likely to reflect a balance between the arrangement that promotes optimal growth, and alternative forms that inhibit movement of DNA originating from such fast-dividing cells. How such differences in fitness arise is a topic for further investigation, as are any regulatory signals and mechanisms affecting the locus’ rate of diversification through phase variation. Previous RNA-seq data found *tvr* locus genes to be unusually highly transcribed in the antisense direction (32), which may be indicative of another moderating influence on SpnIV activity, given the known regulation of *E. coli* RMSs by antisense RNAs (47). Alternatively, signals could be transduced via the unknown recombination mechanism that drives RecA- and TvrR-independent excision of the circular intermediates; were this latter mechanism also to operate at the *ivr* locus, it could upregulate a dual response to any stimulus to which it would be beneficial to trigger a change in RMS specificity. Even in the absence of added exogenous signals, the frequent, carefully orchestrated rearrangements of the SpnIV system provide evidence that even this diverse species exhibits some resistance to complete genomic plasticity.

## DATA AVAILABILITY

Accession codes for the sequence data are listed in Supplementary Table S1.

## Supplementary Material

Supplementary Data
