# Supplementary material for: Excision-reintegration at a pneumococcal phase-variable restriction-modification locus drives within- and between-strain epigenetic differentiation and inhibits gene acquisition
Source: Nucleic Acids Res. 2018 Oct 13;46(21):11438–53. doi: 10.1093/nar/gky906 (PMC6265443; doi:10.1093/nar/gky906)
Supplement: Supplementary Data [file gky906_supplemental_files.zip › RMS_NAR_resub_supplementary_figures_combined_.pdf]

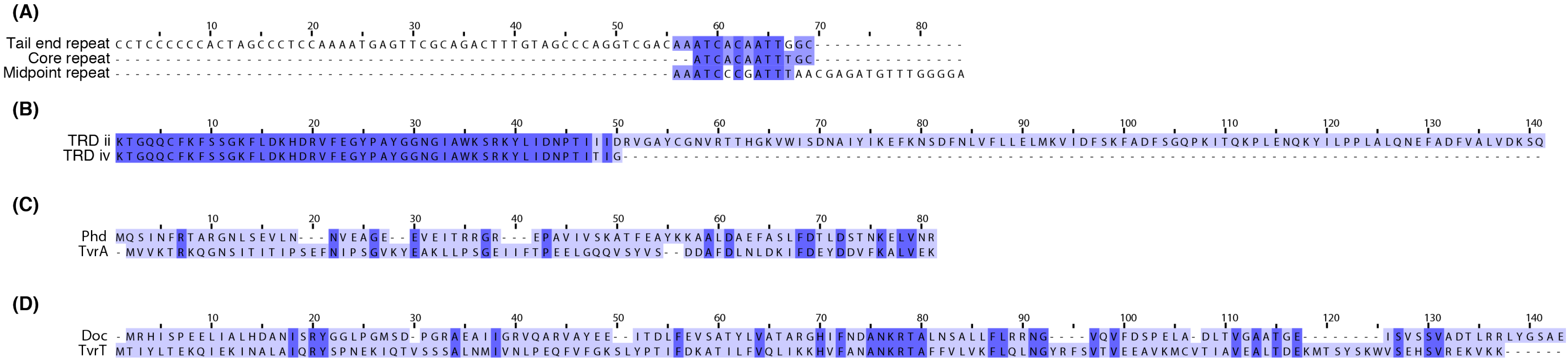

Figure S1 Alignments of *tvr* locus sequences. Columns are shaded according to the level of conservation observed between sequences. (A) Alignment of a midpoint repeat (MPR; marked in green in Fig 1A) and tail-end repeat (TER; marked in purple in Fig 1A) from the *tvr* locus, at which the TvrR recombinase appears to act. These overlap at a shared core sequence, which corresponds with the truncated TER observed in isolate RMV1, coloured in orange in Fig 1A. (B) Alignment of C-terminal TRDs ii and iv. These are highly similar, except the latter is truncated to less than half the length of the former. (C) Alignment of Phd, the anti-toxin from the *doc-phd* locus of *E. coli* phage P1, and TvrA. (D) Alignment of Doc, the toxin from the *doc-phd* locus of *E. coli* phage P1, and TvrT.

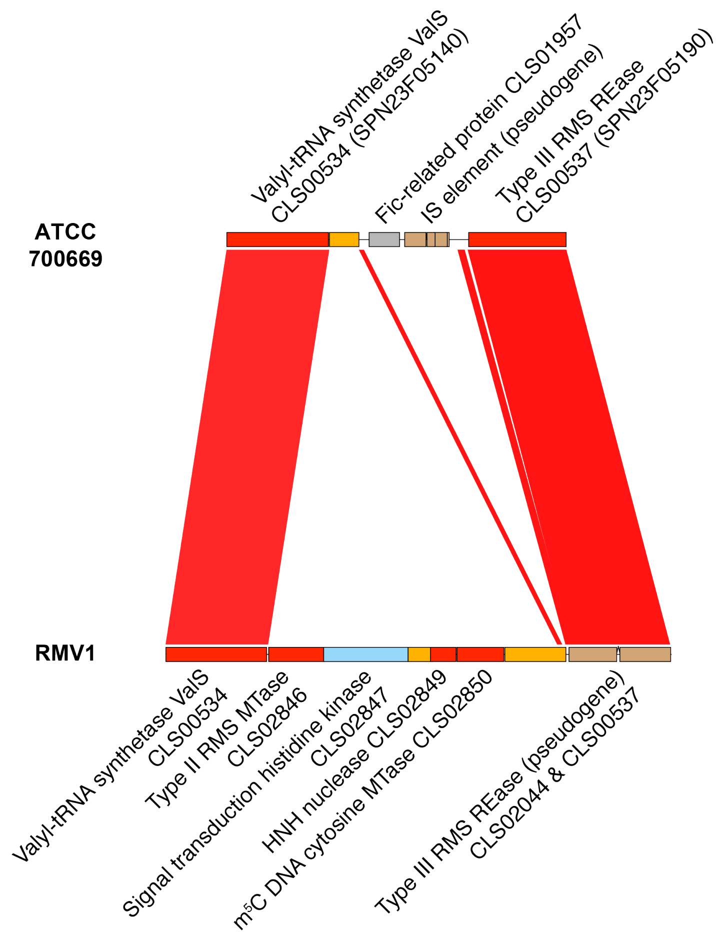

Figure S2 Orphan MTase likely causing methylation of CTBVAG sites in RMV11. This alignment of *S. pneumoniae* ATCC 700669 (accession code FM211187) with *S. pneumoniae* RMV11 spans the region downstream of *valS* (relative to the origin of replication), which encodes the valyl-tRNA synthetase ValS. Boxes represent coding sequences, coloured by functional annotation of the corresponding proteins: red for DNA modification or translation functions, blue for regulatory functions, grey for toxin-antitoxin-like proteins, orange for unknown function, and brown for truncated pseudogene products. The red bands link regions of similar sequence, as identified by BLASTN with default settings, with the intensity of the colour indicating the strength of the match. In *S. pneumoniae*, this locus includes a Type III RMS REase-like protein, which is presumably inactive. Only genes of uncertain function are found between this coding sequence and *valS*. In *S. pneumoniae* RMV11, the same region encodes a gene cluster including an HNH nuclease and two MTases: one predicted

to generate 5-methylcytosine modifications, not typically detected by SMRT sequencing, and one similar to Type II RMSs, expected to generate 6-methyladenosine at a palindromic sequence. As this MTase is not present in any other RMV, it is likely to be responsible for the ^{m6}A modification at the CTBVAG motif. In the *S. pneumoniae* RMV11 genome, the putative Type III REase contains a frameshift mutation, supporting the contention it is non-functional.

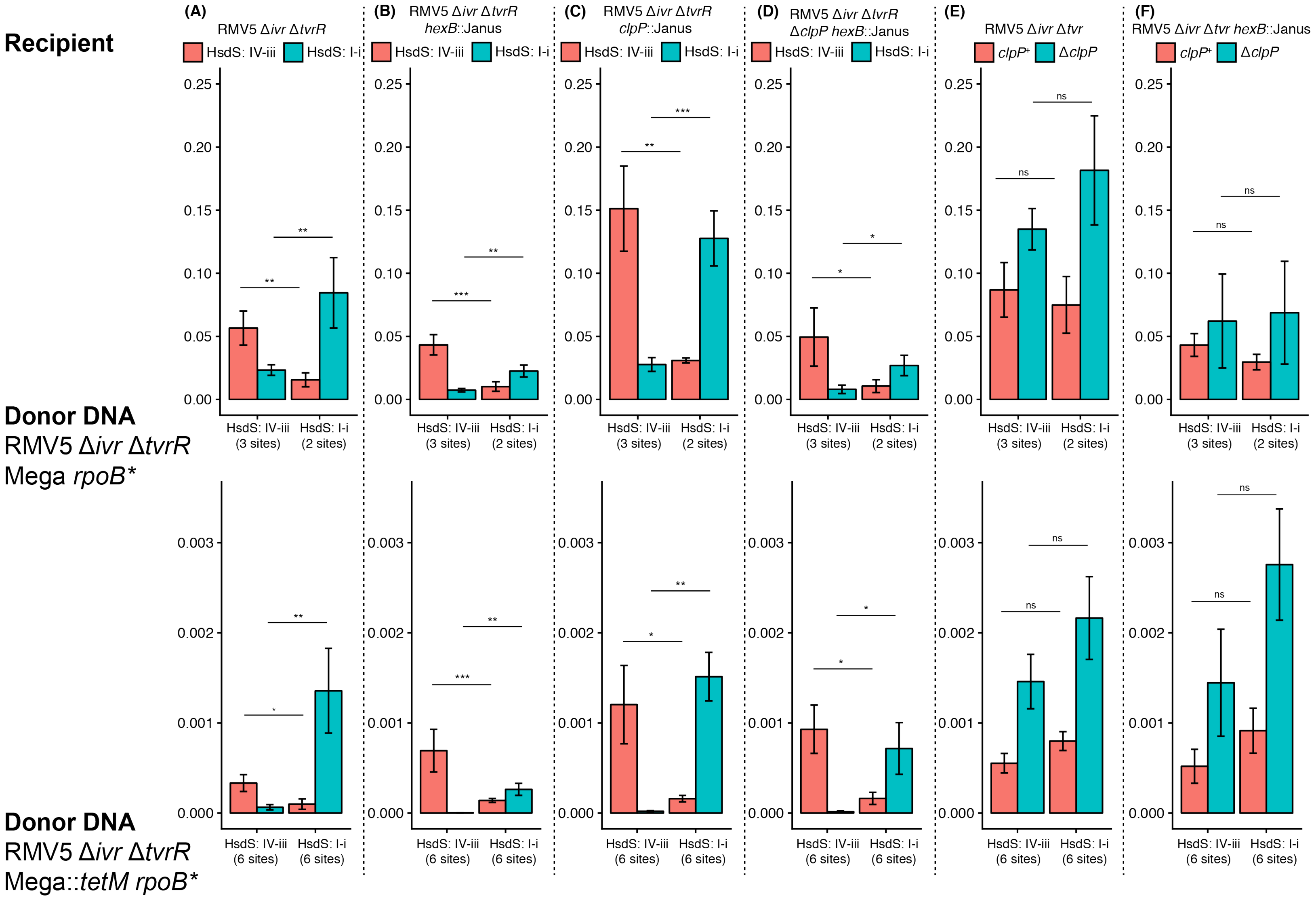

Figure S3 Inhibition of GI import through transformation by the SpnIV RMS. These barcharts quantify the rate of transformation with a GI relative to a rifampicin

resistance single nucleotide polymorphism (SNP). The GI was either the Mega cassette (top row), conferring macrolide resistance, or a derivative of this cassette carrying a *tetM* gene and artificial sequence, containing six recognition sites for the SpnIV HsdS I-i and IV-iii (bottom row). The donor and recipient genotypes were locked through knock out of *tvrR*, and therefore expressed a fixed SpnIV specificity protein composed of the TRDs I-i or IV-iii. All annotated comparisons were performed with the Wilcoxon rank sum test on at least five biological replicates. The error bars show the standard error of the mean. (A) GI acquisition is lower when donor and recipient have a different SpnIV specificity protein, implying the RMS causes self-restriction of the imported GI at unmodified recognition sites. For the recipient expressing the IV-iii HsdS, an approximately five-fold reduction in import was observed for the Mega cassette (which contains three IV-iii motifs) or the Mega:*tetM* GI (which contains six IV-iii motifs). For the recipient expressing the I-i HsdS, the approximately three-fold inhibition of import observed for the Mega cassette (which contains two I-i motifs) increased to an ~22-fold inhibition of import for the Mega::*tetM* GI (which contains six I-i motifs). (B) To test whether there was any dependence on mismatch repair, which could affect the transfer of the SNP causing rifampicin resistance, the experiment was repeated with recipient cells were used in which the *hexB* mismatch repair gene was disrupted with the Janus cassette. The patterns of exchange and fold changes were similar to those in mismatch repair-proficient hosts, although the absolute ratios of GI to SNP import were reduced, due to the increased efficiency of SNP acquisition. Hence the observed differences in ratios between recipients are independent of effects on the SNP recombination rate. (C) To test for the effects of *clpP* knockout, the experiments were repeated in recipients in which the *clpP* gene was disrupted by a Janus cassette. In these experiments, the fold difference between donor DNA with the matching, and non-matching, patterns of modification for the Mega and Mega::*tetM* GIs increased to five and eight fold, and six and sixty fold, for the IV-iii and I-i recipients, respectively. This

indicated the proteolytic action of ClpP had a bigger effect on increasing the rate of transformation with GIs than it did on downregulating the RMS activity. (D) The joint effects of *hexB* and *clpP* disruption were tested. The pattern of GI transfer was again similar to the previous experiments, with the absolute ratio of GI to SNP transfer decreased by the loss of mismatch repair. (E) Testing for dependence on SpnIV activity. In these experiments, the recipient cells lacked the *tvr* locus. Correspondingly, the ratio of GI to SNP import was similar, regardless of the SpnIV specificity protein expressed by the DNA donor. This is consistent with the differences observed in the other panels depending on the self-restricting activity of the SpnIV RMS. The disruption of *clpP* in the recipients was again observed to increase the rate of GI import, independently of the SpnIV specificities, confirming the effects of this mutation were not mediated through this RMS. (F) Testing for dependence on SpnIV activity in recipients lacking a mismatch repair system. These results were similar to those in panel (E), except for the increased rate of SNP acquisition by transformation decreasing the absolute values of the ratios in the experiments with the unmodified Mega cassette.

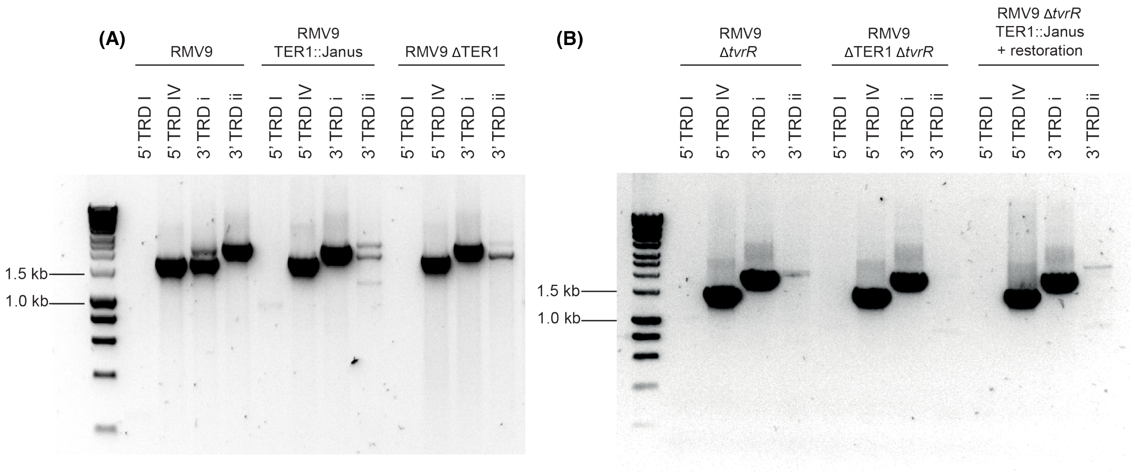

Figure S4 PCR amplifications were performed as in Fig 1B with the fixed primer, *tvrAL*. (A) Reduced shuffling of the TESs is observed when TER1 is replaced by the Janus cassette in RMV9. This confirms that when TER1 is replaced by a Janus cassette or completely removed, the resulting mutants reduce shuffled TESs at a reduced rate. (B) RMV9 Δ*tvrR* shows the almost locked arrangements of TESs as for the other Δ*tvrR* in Fig 1C. The double mutant, RMV9 Δ*tvrR* ΔTER1 showed no detection of circular forms in Fig 3D, and correspondingly did not show the band corresponding to the TES of TRD ii moving downstream of *tvrAT*. The weak band is again observed when the TER is restored. This indicates that the weak variation achieved via the unknown TvrR-independent recombination pathway decreases when a purple repeat is disrupted.

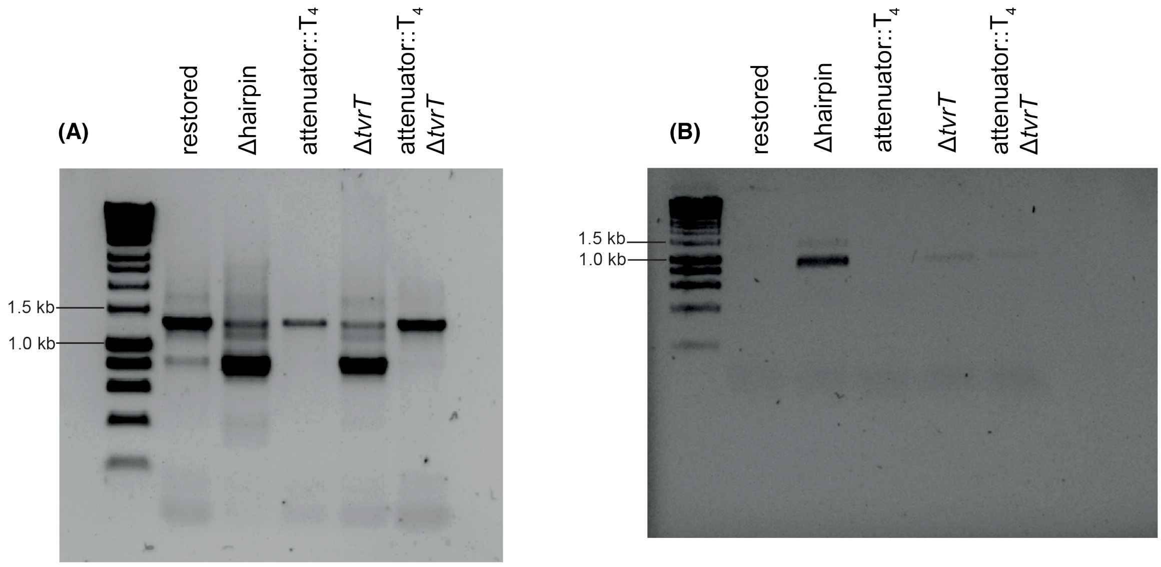

Figure S5 Effect of changes in *tvrR* expression on the prevalence of circular forms excised from the *tvr* locus. (A) Circular forms were detected through PCR amplification with the primers Lcirc and Rcirc. In both the attenuator::T_4 and attenuator::T_4 Δ*tvrT* mutants, in which *tvrR* transcription was reduced, one band was evident. This is consistent with reduced TvrR-mediated excision, resulting in detection only of the form that is more prominent in the wild type background, thereby lowering the rate of TES shuffling. Conversely, the Δhairpin and Δ*tvrT* mutants, in which *tvrR* expression was increased, produced two PCR products. In these lanes, the smaller band was more prominent, likely reflecting elevated levels of both circular forms, with the template for the shorter amplicon present at sufficient levels for this product to outcompete production of the larger one. (B) Detection of transcription from circular forms using cDNA samples. The Δhairpin mutant, in which transcription of *tvrR* was highest, produced two bands in the PCR gel corresponding to the correct sizes of the expected circular forms. This shows that the genes on the circular forms of the *tvr* locus are still actively transcribed, generating *tvrR* transcripts to facilitate reintegration.

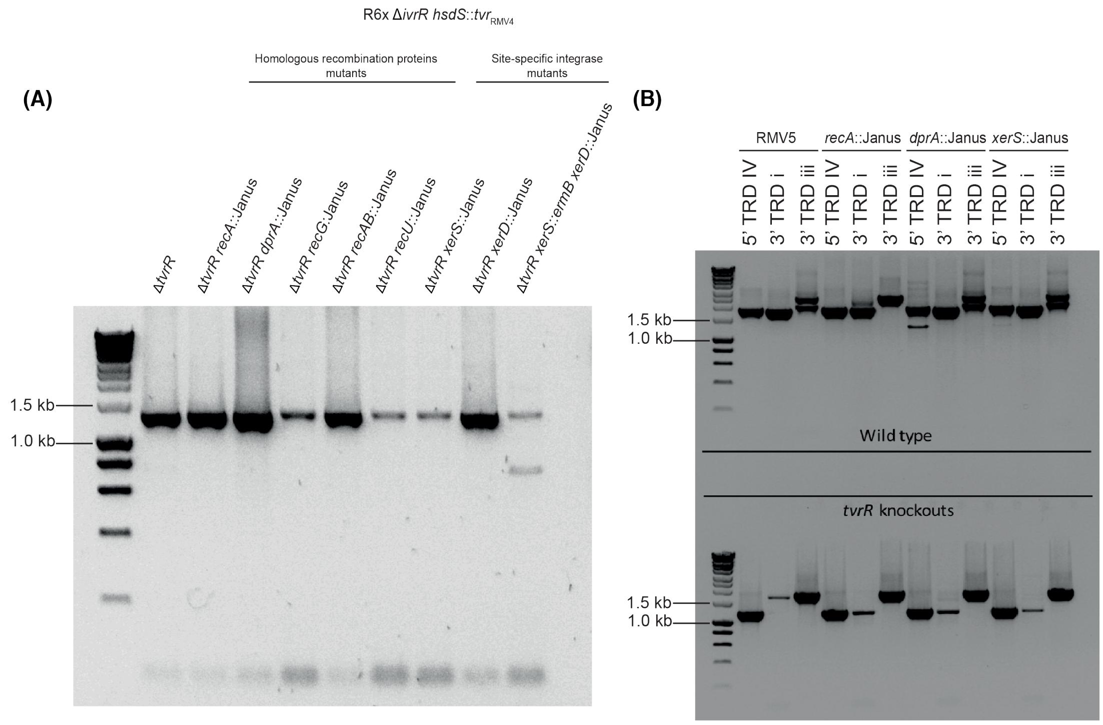

Figure S6 Generation of circular forms is resilient to disruption of multiple recombination pathways. (A) Detection of circular forms in double mutants constructed in a Δ*tvrR* background. Neither disruption of site-specific recombination genes (Δ*tvrR xerS*::Janus, Δ*tvrR xerD*::Janus, Δ*tvrR xerD*::Janus *xerS*::*ermB*), nor of competence genes (Δ*tvrR dprA*::Janus), nor of homologous recombination genes (Δ*tvrR recA*::Janus, Δ*tvrR recU*::Janus, Δ*tvrR rexAB*::Janus, and Δ*tvrR recG*::Janus), prevented detection of circular forms of the *tvr* locus. (B) Continued rearrangement of the *tvr* locus following disruption of recombination pathways. PCR amplifications were performed as in Fig 1B with the fixed primer, tvrAL. Continued shuffling of the TESs within the *tvr* locus is evident when homologous recombination is impaired through disruption of *recA*, competence is impaired through disruption of *dprA*, or chromosomal dimer resolution is impaired through disruption of *xerS*. Although the rate of variation is reduced in the Δ*tvrR* mutant, there is still evidence of TES shuffling in the double mutants (bottom panel), based on the weak band detected with the primer specific to the 3' TES i.

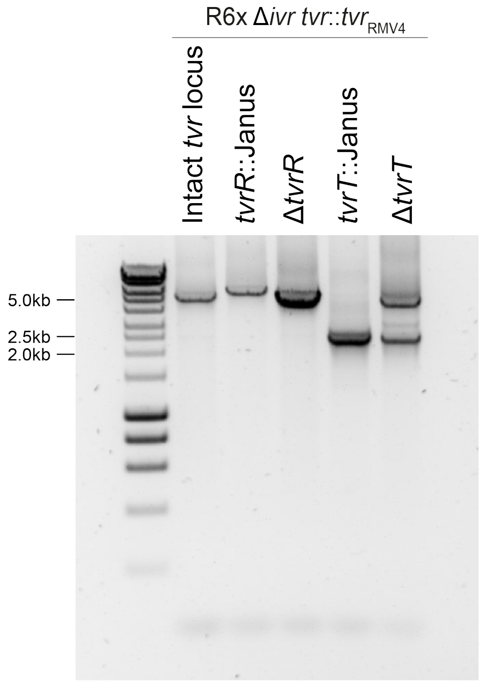

Figure S7 Independence of observed phenotype from inter-repeat distance. This gel shows PCR amplification products generated with primers hsdML and hsdRR using template DNA from R6x mutants carrying the *tvr* locus of RMV4. Insertion of the dual-selectable Janus cassette marker maintains or increases the size of the *tvr* locus; removal of the cassette decreases the locus size by around one kilobase. The stability of the *tvr* locus in the $tvrR^{-}$ genotypes is reflected by the observation of only a single high molecular weight band in the *tvrR*::Janus and Δ*tvrR* genotypes. The high rate at which circular intermediates are excised in the $tvrT^{-}$ genotypes results in shorter bands being observed in both the *tvrT*::Janus and the Δ*tvrT* genotypes. An additional higher molecular weight band, representing the full-length locus, is observed in the Δ*tvrT*, but not the *tvrT*::Janus, lane. This likely reflects the latter having a greater size differential between the full locus, and that from which the circular intermediate has been excised, because the Janus cassette is excised on the circular molecule. Therefore the rate of excision appears to be strongly dependent on whether *tvrR* and *tvrT* are intact, rather than reflecting the deletion of the Janus cassette facilitating more rapid excision when the repeat sequences within the *tvr* locus are brought closer together.

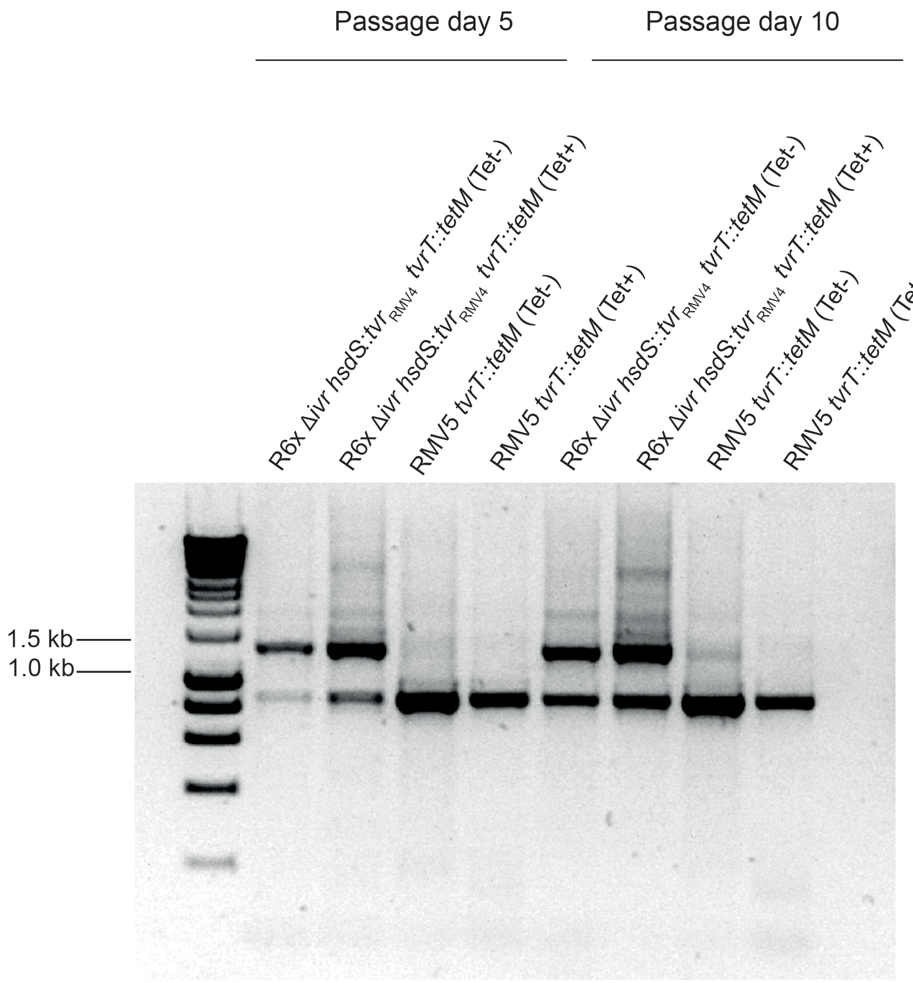

Figure S8 Detection of circular forms using PCR amplification with the primers Lcirc and Rcirc in the *tvrT*::*tetM* mutants harvested after the fifth and tenth days of the passages. "Tet-" denotes those cultures grown in non-selective media, whereas "Tet+" indicates the cells were grown in the presence of tetracycline, to select for cells retaining the *tetM* gene. PCR amplicons corresponding to the expected sizes of the excised circular forms were observed in all genotypes in all conditions.

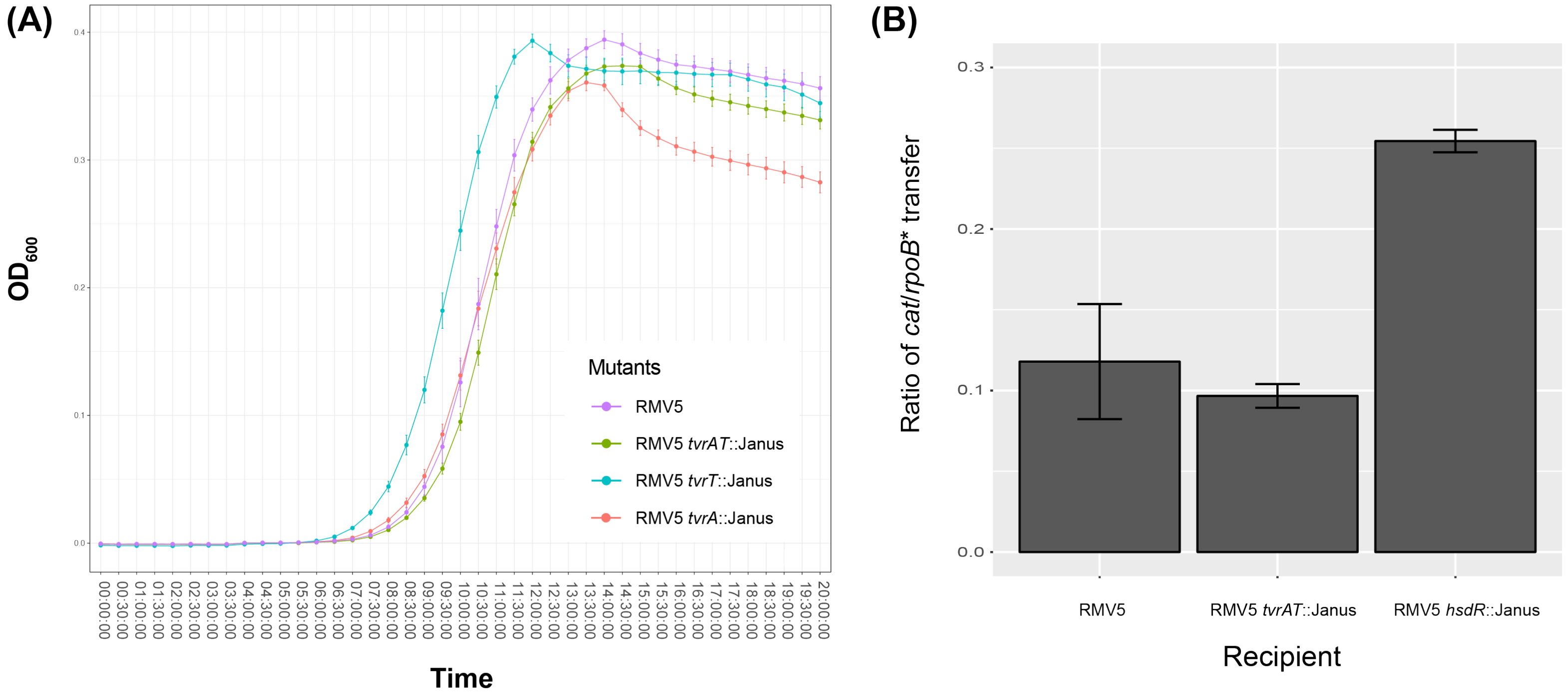

Figure S9 Testing for addictive properties of TvrAT. (A) Growth curves, recorded over 20 h, comparing the *in vitro* fitness of mutants in the *tvrAT* locus. Each point represents the mean OD_{600} measurement, and the error bars represent the standard error of the mean, as calculated from six replicates. Disrupting the *tvrT* gene, encoding a putative toxin, did reduce the lag phase of growth, consistent with TvrAT being an addictive toxin-antitoxin system. However, removal of *tvrAT* did not cause a similar increase in growth rate, despite this mutation eliminating production of the putatively redundant antitoxin as well as the toxin, suggesting alternative effects caused the *tvrT* growth phenotype. Finally,

disruption of *tvrA* did not substantially inhibit logarithmic-phase growth relative to the *tvrAT*::Janus mutant, suggesting the TvrT protein did not have a significant detrimental effect on cell fitness in the absence of the cognate antitoxin during this phase of growth. However, there was an increase in net cell death during stationary phase. (B) Efficiency of *tvr* deletion. Donor genomic DNA from *S. pneumoniae* R6x was generated in which a chloramphenicol acetyltransferase (*cat*) gene was present in place of the *tvr* locus, and the *rpoB* gene contained a mutation conferring rifampicin resistance. Transforming RMV5 recipient cells with this DNA allowed a ratio of chloramphenicol-resistant colonies to rifampicin-resistant colonies to be calculated, which quantified the relative efficiency with which the *tvr* locus was deleted by homologous recombination. Error bars show the standard error of the mean. These data from four replicates demonstrated that disrupting the *tvrAT* genes with a Janus cassette did not significantly affect the efficiency with which the *tvr* locus was deleted, indicating they did not exhibit an addictive effect in this assay. By contrast, disrupting *spnTVRhsdR* significantly increased the efficiency of deletion, by more than two-fold. This is likely a consequence of the *cat* gene, used for deletion, containing a restriction site targeted by this system in the wild-type and *tvrAT*::Janus genotypes, meaning self-restriction kills some transformants that acquire *cat* when the SpnIV HsdR is active. Hence these two experiments jointly demonstrate that the TvrAT system is not addictive during logarithmic phase, when the cells are competent to be transformed, although the proteins could exhibit such an effect during stationary phase.

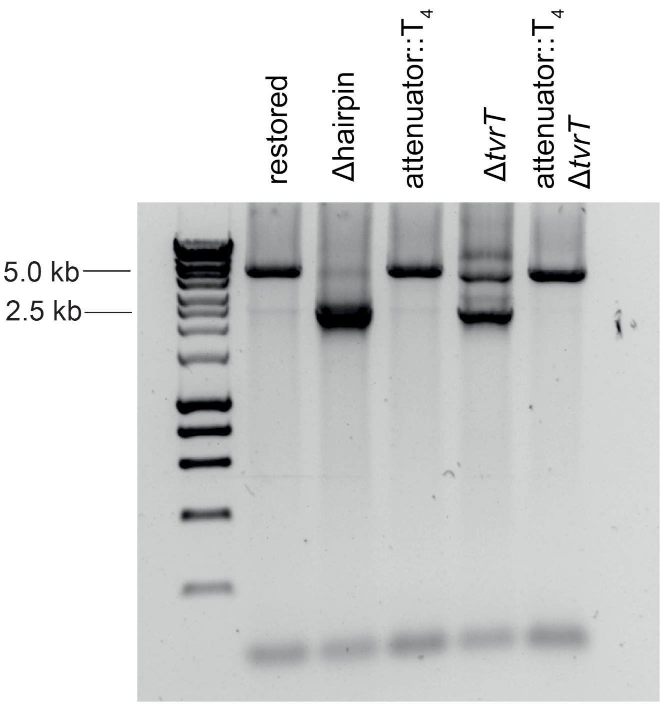

Figure S10 Amplification of the linear *tvr* loci in mutant genotypes, using primers hsdML and hsdRR, demonstrated that the Δhairpin and Δ*tvrT* mutants show an increased prevalence of the shorter *tvr* locus, resulting in amplicons ~2.5 kb in length. This is likely to reflect the faster rates of circular form excision relative to the strain with a restored wild type locus, owing to the mutants' elevated expression of *tvrR*. This suggests production of TvrR is rate-limiting for *tvr* phase variation.

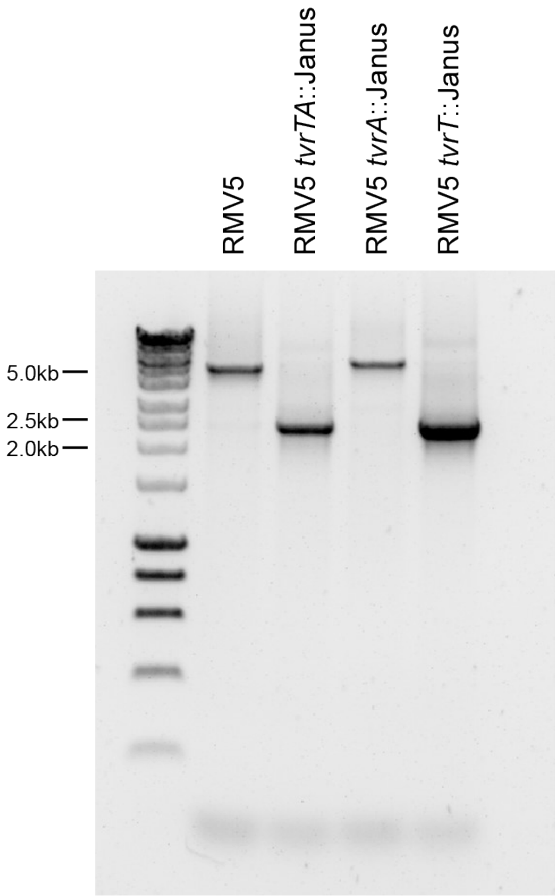

Figure S11 Regulation of *tvr* locus activity by the TvrAT system. PCRs using hsdML and hsdRR amplified the *tvr* locus of RMV5 and mutant derivatives with different Janus cassette insertions into the *tvrAT* locus. Disrupting *tvrT* resulted in amplification of a lower molecular weight band, indicative of frequent excision of circular intermediate molecules. This is consistent with TvrT repressing the activity of TvrR. Disruption of *tvrA* did not have the same effect, demonstrating the phenotype of the *tvrT*::Janus was not a consequence of the cassette insertion itself, and that TvrA and TvrT do not act in conjunction with one another. Disrupting both genes replicates the phenotype of the *tvrT*::Janus mutant, indicating the net effect of the TvrAT proteins in the intact locus is to reduce excision of circular intermediate molecules.

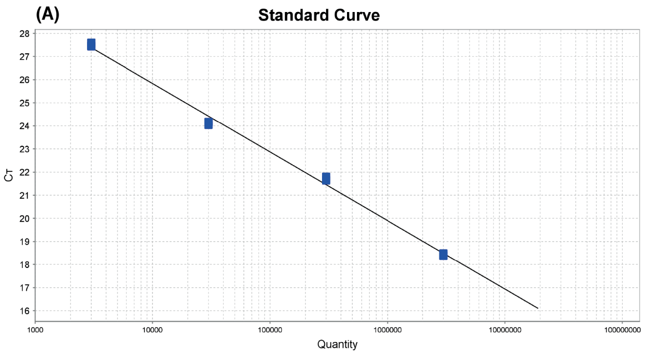

qPCR validation
for the reference gene, *rpoA*

PCR efficiency = 117.4 %
R^2 = 0.996
y-intercept = 37.692
Linear dynamic range = 18.408 - 27.594
Cq variation at lower limit = 0.083 (SD)

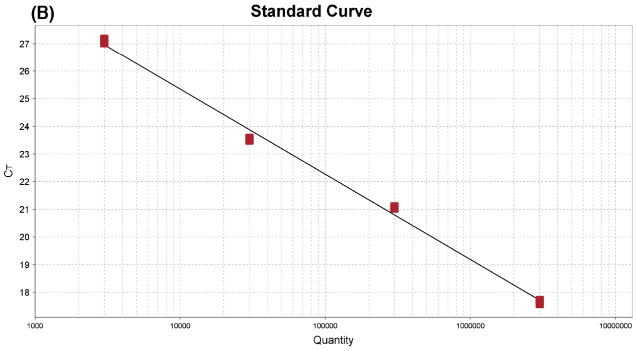

qPCR validation
for the gene of interest, *tvrR*

PCR efficiency = 111.1 %
R^2 = 0.995
y-intercept = 37.677
Linear dynamic range = 17.577 - 27.122
Cq variation at lower limit = 0.081 (SD)

Figure S12 qPCR validation experiments. The standard curves were drawn with Applied Biosystems qRT-PCR software v1.2 using serial dilutions of gDNA for (A) the reference gene, *rpoA*, and (B) the gene of interest, *tvrR*. The values for PCR efficiency, y-intercept, R^2, linear dynamic range and Cq variations at the lowest limit (shown as standard deviation, SD) were calculated and derived from the standard curves.

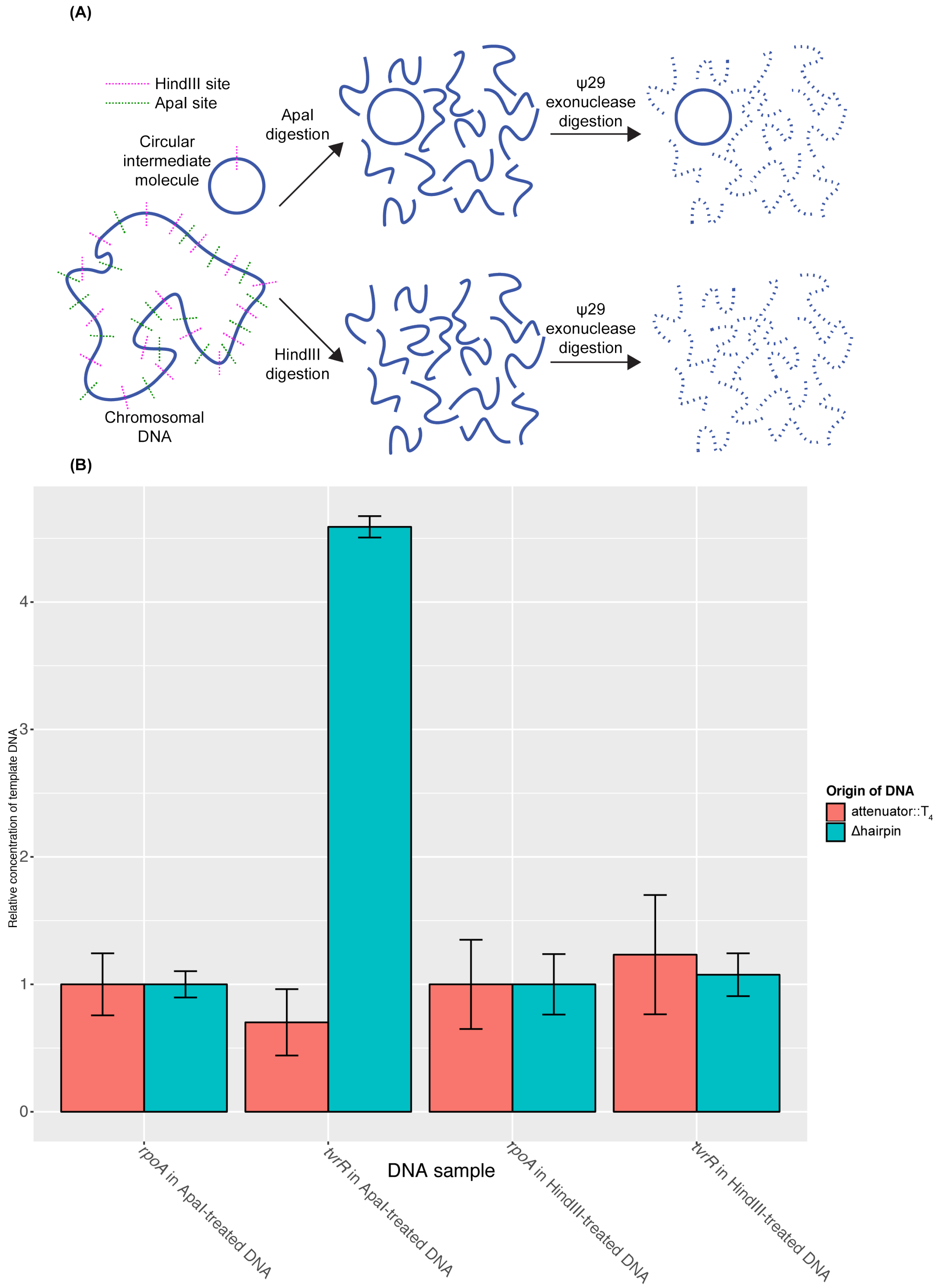

Figure S13 Quantifying circular intermediate molecule formation. The product generated by primers Lcirc and Rcirc was too long for use in accurately quantifying the concentration of circular forms directly, and these transient intermediates were

only present at low concentrations that prevented their isolation at detectable concentrations. Therefore a nuclease-based assay was developed. (A) Description of the assay. REase digestion was first used to selectively linearise circular DNA molecules. ApaI cleaved the chromosome multiple times, but left the circular molecules excised from the *tvr* locus intact. HindIII digested both the chromosome and *tvr* locus circular intermediate molecules. The digest products were then treated with φ29 exonuclease, which digests linear DNA only. Therefore the circular intermediate molecules should only be enriched by this treatment following ApaI treatment. (B) Results of the assay. Quantitative RT-PCR was used to measure the concentration of *tvrR*, found on both the circular intermediate molecules and the chromosome, relative to *rpoA*, found only on the chromosome, following the process described in panel (A). Three technical replicates were performed on each of two biological replicates from each of *S. pneumoniae* R6x Δhairpin and attenuator::T_4 mutants, expected to produce high and low levels of circular intermediate molecules, respectively. The concentration of *tvrR* DNA was approximately five-fold greater in the samples from the Δhairpin mutant, relative to the attenuator::T_4 mutant, when ApaI was used to initially digest the samples. However, no such difference was observed when HindIII was used to digest the samples, indicating efficient cleavage of the circular intermediate molecules in the samples from both mutants.

(A)

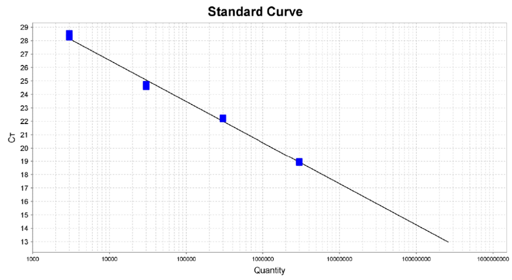

For qPCR validation
for the reference gene, *rpoA*

PCR efficiency = 111.7 %
R^2 = 0.994
y-intercept = 38.821
Linear dynamic range = 18.932- 28.353
Cq variation at lower limit = 0.076 (SD)

(B)

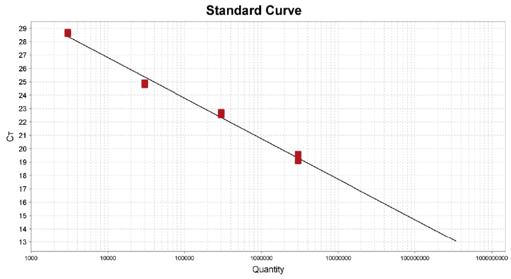

For qPCR validation
for the gene of interest, *tvrR*

PCR efficiency = 113.7 %
R^2 = 0.990
y-intercept = 38.946
Linear dynamic range = 19.273 - 28.639
Cq variation at lower limit = 0.083 (SD)

Figure S14 qPCR validation for the experiment described in Supplementary Fig S13, displayed as described in Supplementary Fig S12.

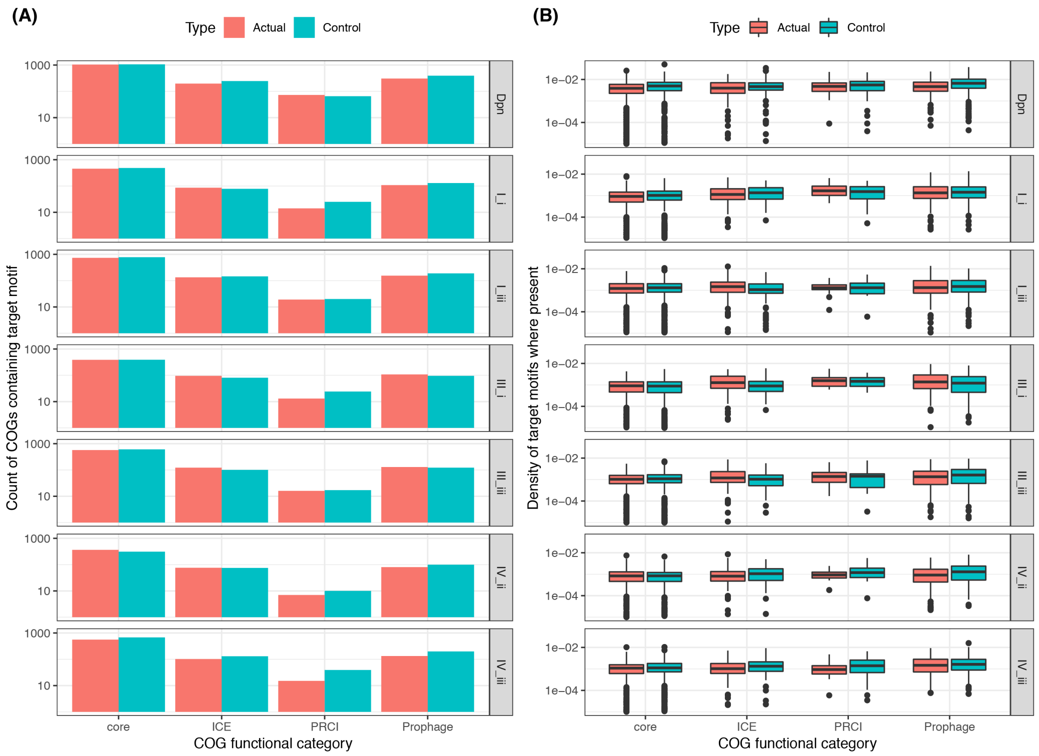

Figure S15 Analysis of RMS motif distribution by clusters of orthologous genes (COGs). These were divided into the core COGs (*n* = 1194), and three categories of mobile genetic element: integrative and conjugative elements (ICE, *n* = 355), prophage-related chromosomal islands (PRCIs; *n* = 142), and prophage (*n* = 590). These were searched for the relevant motif, and a control motif in which the two halves of the bipartite motif were reversed but not complemented; for Dpn, these sites were GATC and TCGA, respectively. (A) Number of COGs containing at least one instance of the actual and control motifs in each of the functional categories. The only discernable difference in these motifs' distribution is slighter fewer PRCI COGs contained some SpnIV motifs than expected from the control motif distribution. (B) The density of motifs was compared across the functional categories for all COGs containing motifs at a density greater than 10^{-5} bp^{-1}. Little consistency was observed in the small differences between the actual and control motifs.

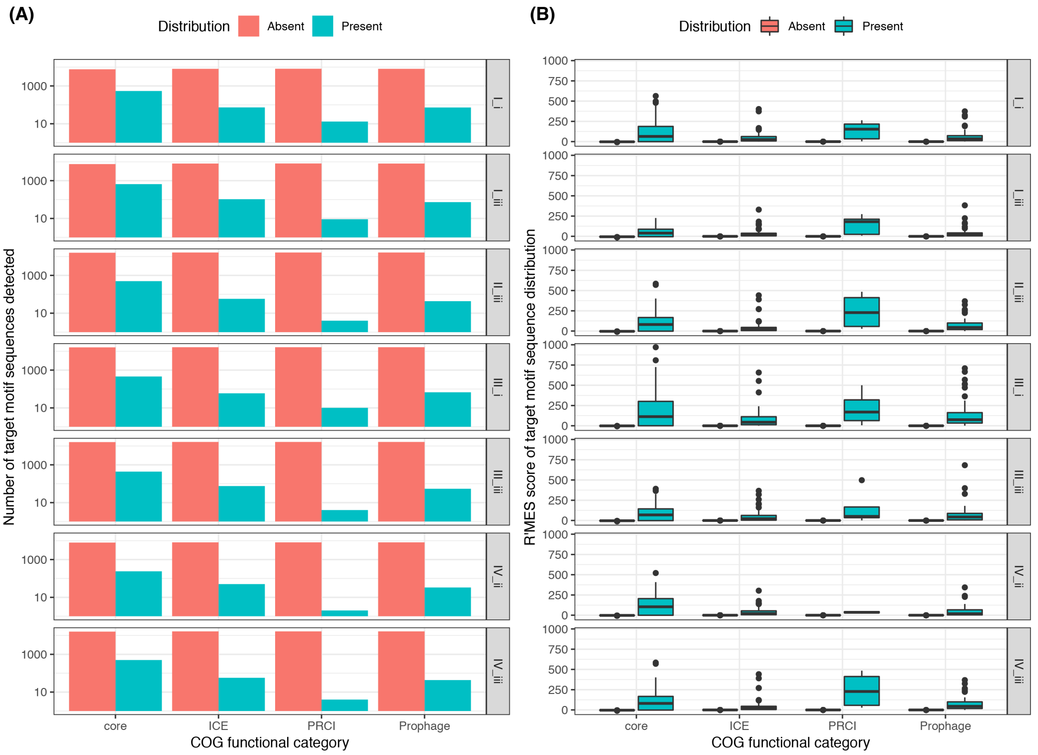

Figure S16 Analysis of SpnIV RMS motif sequence distribution across COG functional categories using R'MES with a Gaussian model, which is designed to estimate the incidence of motifs expected to be common. These graphs show the distribution aggregated across the specific sequences matching the degenerate motif recognised by the SpnIV RMSs. The dataset sizes varied by HsdS specificity: motifs with six unspecified bases corresponded to at least 4,096 different sequences, while motifs with seven unspecified bases corresponded to at least 16,384 different sequences. (A) Number of motif sequences present, at least once, in each of the four functional categories described in Fig S15. Notably fewer sequences are present in the PRCI COGs, although these are less numerous than the others. (B) Boxplot showing the R'MES scores, quantifying the extent to which the motif sequences are over- (positive scores) or under-represented (negative scores), relative to the R'MES Gaussian model of expected frequencies. Results are split for those motif sequences found to be absent or present in each of the functional categories. These results show all the motifs are expected to be sufficiently rare that none of them have

negative scores of a large magnitude, even when completely absent from a functional category. This suggests the results in Fig S17 may be more informative. Motif sequences present in ICE and prophage occur at expected frequencies, with few large positive scores. Despite the smaller proportion of motif sequences observed in PRCIs, those that are present are associated with higher scores, likely representing lower likelihood of them being found in this smallest of the four datasets. Those sequence motifs present in the core are also associated with higher scores, although this may represent the higher conservation of the core COGs, relative to the more variable mobile elements, meaning detected motifs are preserved between the representatives of a single COG. The corresponding scores for the single Dpn motif, GATC, were -941.6, -118.1, -55.3 and -118.6 in the core, ICE, PRCI and prophage COGs, respectively. This represents a substantial depletion of the motif, across all categories, which is strongest in the pneumococcal core genome.

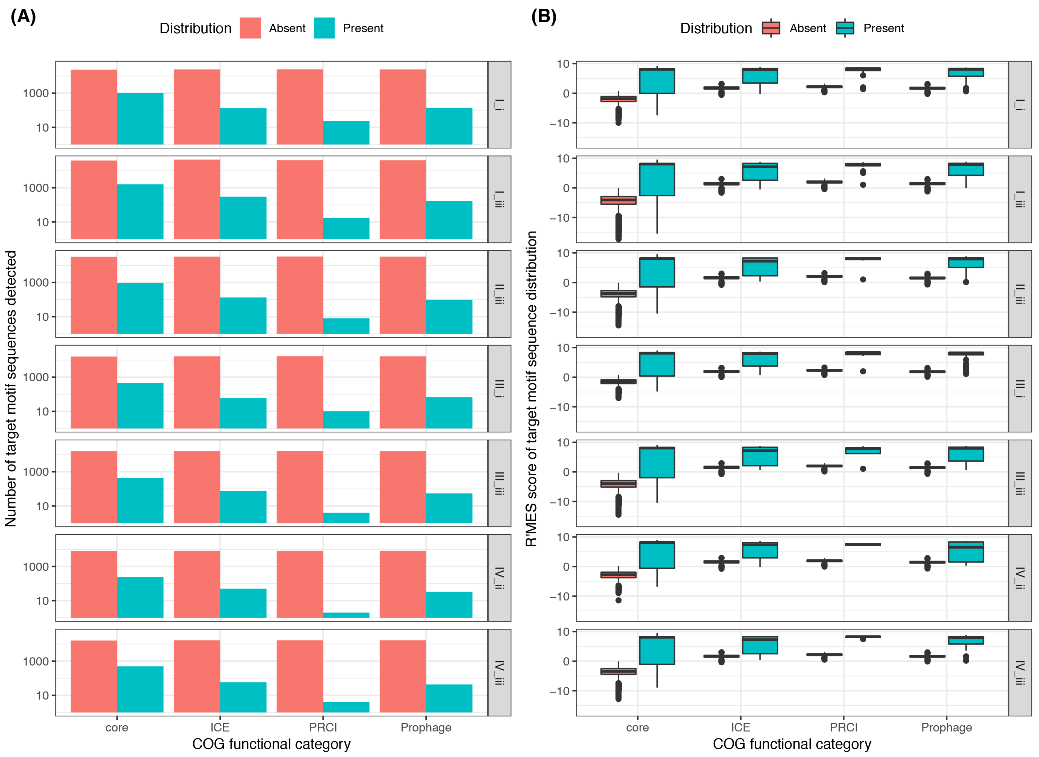

Figure S17 Analysis of SpnIV RMS motif sequence distribution across COG functional categories using R'MES with a compound Poisson model, which is designed to estimate the incidence of motifs expected to be rare. These graphs show the distribution aggregated across the specific sequences matching the degenerate motif recognised by the SpnIV RMSs. (A) Number of motif sequences present, at least once; this is identical to panel A of Fig S16. (B) Boxplot showing the R'MES scores, quantifying the extent to which the motif sequences are over- (positive scores) or under-represented (negative scores), relative to the R'MES compound Poisson model of expected frequencies. Results are split for those motif sequences found to be absent or present in each of the functional categories. These results again show all the motifs are expected to be sufficiently rare that none of them have large negative scores, even when completely absent from a functional category. The score for motifs found to be present in these different COG datasets follow the pattern described in Fig S16. The corresponding scores for the single Dpn motif, GATC, were sufficiently depleted in the core, ICE, PRCI and prophage COGs that

the model could not be fitted with a finite score. This confirms the GATC sequence is significantly rarer in the pangenome than expected by chance, suggesting evolution influenced by restriction avoidance.

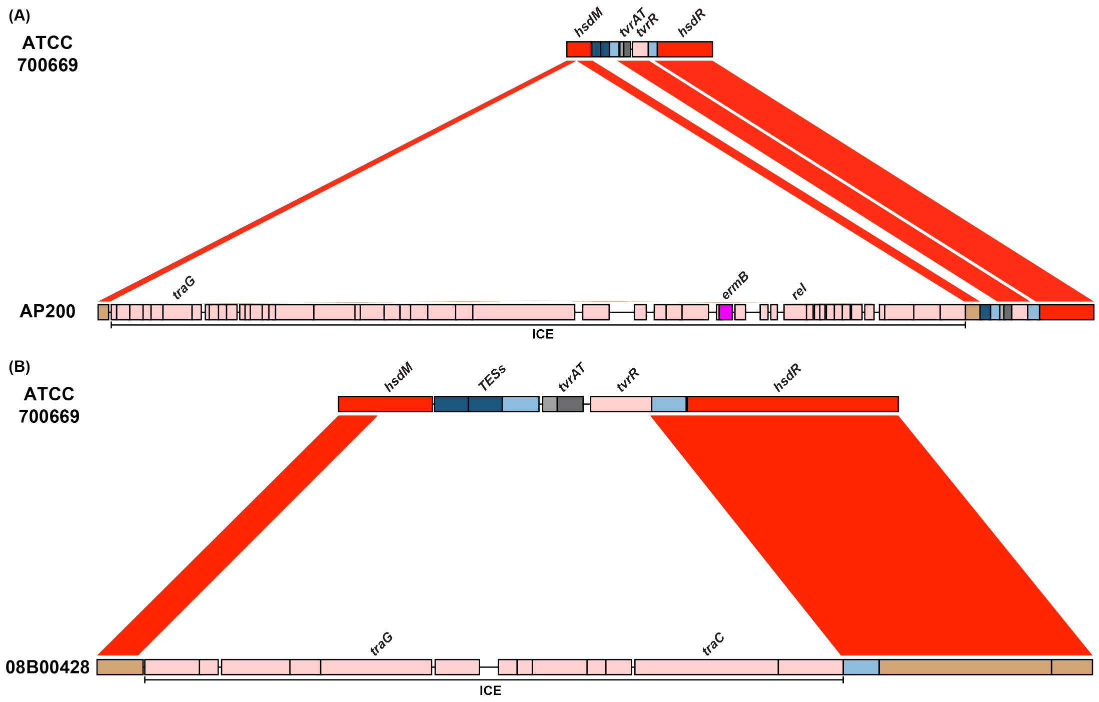

Figure S18 Integrative and conjugative elements (ICEs) inserted into the *tvr* locus *hsdM* gene. Each panel compares the intact *tvr* locus of *S. pneumoniae* ATCC 700669 (accession code FM211187) with an orthologous locus into which an ICE has inserted. ICE coding sequences are coloured pink; the *tvr* locus coding sequences are coloured as in Fig 1. Red bands link regions of similar sequence. (A) Comparison with *S. pneumoniae* AP200 (accession code CP002121). A ~52 kb ICE, carrying the *ermB* macrolide resistance gene (highlighted in purple), is inserted into the *spnTVRhsdM* gene. (B) Comparison with *S. pneumoniae* 08B00428 (accession code CFEZ01000000), isolated from the Maela refugee camp. An ~11 kb ICE is inserted into the *spnTVRhsdM* gene, the 3' segment of which has been lost, along with *tvrATR*. A nonsense mutation in the *spnTVRhsdR* gene is predicted to truncate the REase product.
