# Supplementary material for: Excision-reintegration at a pneumococcal phase-variable restriction-modification locus drives within- and between-strain epigenetic differentiation and inhibits gene acquisition
Source: Nucleic Acids Res. 2018 Oct 13;46(21):11438–53. doi: 10.1093/nar/gky906 (PMC6265443; doi:10.1093/nar/gky906)
Supplement: Supplementary Data [file gky906_supplemental_files.zip › RMS_NAR_resub_supplementary_tables.docx]

**Supplementary Table S1** Genotypes of strains used in this study.

| ***S. pneumoniae***  **strains** | **Description of original genotype and list of derived mutants** | **Accession code for ENA** |
| --- | --- | --- |
| R6x | Unencapsulated highly transformable laboratory strain derived from D39 |  |
|  | R6x *rpsL** (described in Apagyi *et al* 2018) |  |
|  | R6x *rpsL** Δ*ivr* (described in Apagyi *et al* 2018) |  |
|  | R6x *rpsL** Δ*ivr hsdS::tvr*_RMV4_ |  |
|  | R6x *rpsL** Δ*ivr hsdS::tvr*_RMV4_ Δ*tvrR* |  |
|  | R6x *rpsL** Δ*ivr hsdS::tvr*_RMV4_ Δ*tvrR recA::*Janus |  |
|  | R6x *rpsL** Δ*ivr hsdS::tvr*_RMV4_ Δ*tvrR dprA::*Janus |  |
|  | R6x *rpsL** Δ*ivr hsdS::tvr*_RMV4_ Δ*tvrR recG::*Janus |  |
|  | R6x *rpsL** Δ*ivr hsdS::tvr*_RMV4_ Δ*tvrR recU::*Janus |  |
|  | R6x *rpsL** Δ*ivr hsdS::tvr*_RMV4_ Δ*tvrR recAB::*Janus |  |
|  | R6x *rpsL** Δ*ivr hsdS::tvr*_RMV4_ Δ*tvrR xerS::*Janus |  |
|  | R6x *rpsL** Δ*ivr hsdS::tvr*_RMV4_ Δ*tvrR xerD::*Janus |  |
|  | R6x *rpsL** Δ*ivr hsdS::tvr*_RMV4_ Δ*_Δ_tvrR ccrB::*Janus |  |
|  | R6x *rpsL** Δ*ivr hsdS::tvr*_RMV4_ *tvrT::*Janus |  |
|  | R6x *rpsL** Δ*ivr hsdS::tvr*_RMV4_ *tvrAT::*Janu*s* |  |
|  | R6x *rpsL** Δ*ivr hsdS::tvr*_RMV4_ *tvrT::tetM* |  |
|  | R6x *rpsL** Δ*ivr hsdS::tvr*_RMV4_ Δ*tvrT* |  |
|  | R6x *rpsL** Δ*ivr hsdS::tvr*_RMV4_ Δ*tvrT* attenuator*::*T_4_ |  |
|  | R6x *rpsL** Δ*ivr hsdS::tvr*_RMV4_ Δhairpin |  |
|  | R6x *rpsL** Δ*ivr dpnI::*Janus |  |
|  | R6x *rpsL** Δ*ivr dpnII* |  |
|  | R6x *rpsL** Δ*ivr dpnII dpnA::*Janus |  |
|  | R6x *rpsL** Δ*ivr rpoB** Mega  R6x *rpsL** Δ*ivr hsdS::cat rpoB** Mega |  |
| RMV1 | Sequence cluster 10; type II RMS locus LK020705; SpnIV TRDs I, IV, iv |  |
|  | RMV1 *rpsL** |  |
|  | RMV1 *rpsL** Δ*tvrR* (TRDI-iv) | ERS1681518 |
|  | RMV1 *rpsL** *tvr*::*cat* | ERS2630969 |
| RMV2 | Sequence cluster 3; SpnIV TRDs II, III, i |  |
|  | RMV2  *rpsL** |  |
|  | RMV2  *rpsL** Δ*tvrR* |  |
| RMV3 | Sequence cluster 1; type II RMS loci LK020705 and LK020708; SpnIV TRDs I, IV, i, iii |  |
|  | RMV3  *rpsL** |  |
|  | RMV3  *rpsL** Δ*tvrR* (TRD I-iii) | ERS1681519 |
| RMV4 | Sequence cluster 5; SpnIV TRDs I, IV, i, iii |  |
|  | RMV4  *rpsL** |  |
|  | RMV4  *rpsL** Δ*tvrR* (TRD I-i) | ERS1681526 |
| RMV5 | Sequence cluster 6; SpnIV TRDs I, IV, i, iii |  |
|  | RMV5  *rpsL** |  |
|  | RMV5  *rpsL* ΔtvrR* (TRDIV-iii) |  |
|  | RMV5  *rpsL** Δ*tvrR* (TRDI-i) | ERS1681525 |
|  | RMV5  *rpsL** Δ*tvrR* (TRDIV-iii) Δ*ivr* |  |
|  | RMV5  *rpsL** Δ*tvrR* (TRDI-i) Δ*ivr* |  |
|  | RMV5  *rpsL** Δ*tvrR* (TRDIV-iii) Δ*ivr hexB*::Janus |  |
|  | RMV5  *rpsL** Δ*tvrR* (TRDI-i) Δ*ivr hexB*::Janus |  |
|  | RMV5  *rpsL** Δ*tvrR* (TRDIV-iii) Δ*ivr clpP::*Janus |  |
|  | RMV5  *rpsL** Δ*tvrR* (TRDI-i) Δ*ivr clpP::*Janus |  |
|  | RMV5  *rpsL** Δ*tvrR* (TRDIV-iii) Δ*ivr clpP::*Janus  *hexB*::Janus |  |
|  | RMV5  *rpsL** Δ*tvrR* (TRDI-i) Δ*ivr clpP::*Janus  *hexB*::Janus |  |
|  | RMV5  *rpsL** Δ*tvrR* (TRDIV-iii) *rpoB** Mega |  |
|  | RMV5  *rpsL** Δ*tvrR* (TRDI-i) *rpoB** Mega |  |
|  | RMV5  *rpsL** Δ*tvrR* (TRDIV-iii) *rpoB** Mega::*tetM* |  |
|  | RMV5  *rpsL** Δ*tvrR* (TRDI-i) *rpoB** Mega::*tetM* |  |
|  | RMV5  *rpsL* recA::*Janus |  |
|  | RMV5  *rpsL* dprA::*Janus |  |
|  | RMV5  *rpsL* xerS::*Janus |  |
|  | RMV5  *rpsL** Δ*tvrR* (TRDI-i) *recA::*Janus |  |
|  | RMV5  *rpsL** Δ*tvrR* (TRDI-i) *dprA::*Janus |  |
|  | RMV5  *rpsL** Δ*tvrR* (TRDI-i) *xerS::*Janus |  |
|  | RMV5  *rpsL* tvrT::tetM* |  |
|  | RMV5  *rpsL* tvrA*::Janus |  |
|  | RMV5  *rpsL* tvrAT*::Janus |  |
|  | RMV5  *rpsL* tvrT*::Janus |  |
|  | RMV5  *rpsL* hsdR*::Janus |  |
| RMV6 | Sequence cluster 15; SpnIV TRDs II, III, i, iii |  |
|  | RMV6  *rpsL** |  |
|  | RMV6  *rpsL** Δ*tvrR* (TRDII-iii) | ERS1681521 |
|  | RMV6  *rpsL**Δ*tvrR* (TRDIII-i) | ERS1681524 |
| RMV7 | Sequence cluster 15; SpnIV TRDs II, III, i, iii |  |
|  | RMV7  *rpsL** |  |
|  | RMV7  *rpsL* tvrR::*Janus (TRDIII-iii) | ERS2478543 |
|  | RMV7  *rpsL** Δ*tvrR* (TRDIII-i) | ERS1681523 |
| RMV8 | Sequence cluster 14; SpnIV TRDs I, IV, i, ii |  |
|  | RMV8  *rpsL** |  |
|  | RMV8  *rpsL** Δ*tvrR* (TRDIV-ii) | ERS1681527 |
|  | RMV8  *rpsL** Δ*tvrR* (TRDI-i) | ERS2478544 |
| RMV9 | Sequence cluster 14; SpnIV I, IV, i, ii |  |
|  | RMV9  *rpsL** |  |
|  | RMV9  *rpsL* tvrT::*Janus |  |
|  | RMV9  *rpsL* tvrAT::*Janus |  |
|  | RMV9  *rpsL** Δ*tvrR* (TRDI-ii) |  |
|  | RMV9  *rpsL** Δ*tvrR* (TRDIV-i) | ERS2478546 |
|  | RMV9  *rpsL** P1*::*Janus |  |
|  | RMV9  *rpsL** ΔTER1 |  |
|  | RMV9  *rpsL** ΔTER1 Δ*tvrR* (TRDI-ii) |  |
| RMV10 | Sequence cluster 9; type II RMS locus LK020709; SpnIV TRDs I, i | ERS2478541 |
| RMV11 | Sequence cluster 9; type II RMS locus LK020709 and LK020710; SpnIV TRDs I, i | ERS2478542 |

**Supplementary Table S2** Sequences of oligonucleotides used in this study.

| **Name of oligonucleotide** | **Sequence** | | **Purpose in this work** | |  |
| --- | --- | --- | --- | --- | --- |
| hsdML | gcggatggtttaagtttgga | | Checking rearrangements of *tvr* | |  |
| tvrAL | aatcaccattacgattccaagtgaattt | | Checking rearrangements of *tvr* | |  |
| hsdRR | accaaaagtgcctgtctacg | | Checking rearrangements of *tvr* | |  |
| Lcirc | aacaaatcattaccgtaattacaatggc | | Detecting circular forms | |  |
| Rcirc | aattgctggtgtagagattcgc | | Detecting circular forms | |  |
| Trd_I_R_V2 | ttgtactaccaacagctcccgataa | | Checking rearrangements of *tvr* | |  |
| Trd_II_R_v4 | tgcttaacaaatccagctcactt | | Checking rearrangements of *tvr* | |  |
| Trd_III_R_v1 | ttcaatataaaactgccatc | | Checking rearrangements of *tvr* | |  |
| Trd_IV_R_v2 | atttatctctgttgaagttttagtcag | | Checking rearrangements of *tvr* | |  |
| Trd_i_R_v1 | ctcctcactaaacaactcatctga | | Checking rearrangements of *tvr* | |  |
| Trd_ii_R_v3 | tcttacattaccacaatatgcacc | | Checking rearrangements of *tvr* | |  |
| Trd_iii_R_v2 | tccaaatgctgtatctacattcca | | Checking rearrangements of *tvr* | | |
| Trd_iv_R_v1 | gccaattgtgatagtagg | | Checking rearrangements of *tvr* | |  |
| qRT_tvrRR | gaattttcgtccgagcgtca | | Transcriptional analysis | |  |
| qRT_tvrRL | caggtatgcgtgtaggggag | | Transcriptional analysis | |  |
| qRT_rpoAR | cacgagcaggttccacttga | | Transcriptional analysis | |  |
| qRT_rpoAL | tggtcgtggatatgtacctgc | | Transcriptional analysis | |  |
| tvrR_inf_UP_R_apaI | gggggcccaatttctcactttcttattca | | Removing *tvrR* | |  |
| tvrR_Inf_UP _R_bamHI | ttggatccaatttctcactttcttattca | | Removing *tvrR* | |  |
| tvrR_inF_Down_F_BamHI | ttggatccttgaggagttatttagcaaatt | | Removing *tvrR* | |  |
| tvrR_Inf_Down_R | cttttaagagatgaatttttggtgtga | | Removing *tvrR* | |  |
| Mega_F | aagggccccttatttgaaagacttttgac | | Amplifying Mega cassette | |  |
| Mega_R | ggtctagatagggccttgtttacagacta | | Amplifying Mega cassette | |  |
| rpoB_L | cgygarcgbatgtcngtwca | | Introducing a SNP into *rpoB* (Apagyi et al, 2018) | |  |
| rpoB_R | tcrtcngcwgtyarccaaac | | Introducing a SNP into *rpoB* (Apagyi et al, 2018) | |  |
| rpsLL | gggacgtgctgacaaatgtt | | Introducing a SNP into *rpsL* (Apagyi et al, 2018) | |  |
| rpsLR | gaagcagctgtaccacgttt | | Introducing a SNP into *rpsL* (Apagyi et al, 2018) | |  |
| tvrAT_KO_UP_V1_apaI | ttgggcccctgatttcctcctttgaagta | | Removing *tvrAT* | |  |
| tvrA_KO_UP_V3_apaI | aagggccctcatttttccaccaaagcttt | | Removing *tvrT* | |  |
| tvrT_BamHI_L | tggatcccctagtatgctggatttgaat | | Removing *tvrAT* /*tvrT* | |  |
| tvrT_ApaL_L | ttgggcccccctagtatgctggatttgaat | | Removing *tvrAT* /*tvrT* | |  |
| Terminator_Down_over_F | tttttttttattgatgaataagaaagtgag | | Adding four extra T bases into the attenuator site | |  |
| Terminator_Up_over_R | cactttcttattcatcaataaaaaaaaagagacaatatcagtttctgcat | | Adding four extra T bases into the attenuator site | |  |
| Terminaotr_UP_R_BamHI | aaggatccgttcatttattttcttgtgct | | Removing the potential hairpin structure | |  |
| Terminator_Down_F_BamHI | aaggatccctttttattgatgaataagaaag | | Removing the potential hairpin structure | |  |
| DpnII_KO_F_UP | aatcagacttcagcagagcatcctg | | Amplifying *dpnII* from RMV2  Removing *dpnI* system from R6x strains | |  |
| DpnII_KO_R_UP_Apal | aagggcccgcttttaattataccatag | | Removing *dpnI* system from R6x strains | |  |
| DpnII_KO_F_Down_BamHI | aaggatccgtccaattcaattagatggg | | Removing *dpnI* system from R6x strains | |  |
| DpnII_KO_R_Down | cctcttgatcaacatctttaccaaagg | | Amplifying *dpnII* from RMV2  Removing *dpnI* system from R6x strains | |  |
| dpnA_KO_UP_F | cctgtgtgatggatgtggatatcacc | | Removing *dpnA* from *dpnII* | |  |
| dpnA_KO_UP_R_apaI | aagggccctggatcagcaaaaatcatatc | | Removing *dpnA* from *dpnII* | |  |
| dpnA_KO_Down_F_BamHI | ccaagcttagtggcactacgggtgttgtt | | Removing *dpnA* from *dpnII* | |  |
| dpnA_KO_Down_R | tctcgtaaattattcttagccttata | | Removing *dpnA* from *dpnII* | |  |
| hsdLL | cgcaaggaagctggtattaca | | Removing *ivr* | |  |
| hsdRR | caatctgaagacctagaaccttgct | | Removing *ivr* | |  |
| Tvr_locus_KO_UP_F | acaacctttgactacgcctatacacc | | Removing t*vr* | |  |
| Tvr_locus_KO_UP_Down_Apal | aagggcccatcttcccttttctttagtt | | Removing t*vr* | |  |
| Tvr_locus_KO_DOWN_F_EcoRV | aagatatcattctaaagtgattgccatgc | | Removing t*vr* | |  |
| Tvr_locus_KO_DOWN_R | aagcccttcttgctaagaacgacattc | | Removing t*vr* | |  |
| TetM_Pro_F_ApaI | ttgggccctaatcacgtactctctttgat | | Amplifying *tetM* | |  |
| TetM_Pro_R_BamHI | ttggatccgtgattttcctccattcaaaa | | Amplifying *tetM* | |  |
| Janus_F | ttgggcccccgtttgatttttaatggataatgtg | | Amplifying Janus | |  |
| Janus_R_V2 | atggatcccctttccttatgcttttggacg | | Amplifying Janus | |  |
| JanusR2_EcoRV | Atgatatccctttccttatgcttttggacg | | Amplifying Janus | |  |
| Janus R_Up_HindIII | ggaagcttgatgttgctgtctcccaggtc | | Inserting *cat* into Janus | |  |
| Janus F_Down_XbaI | ggtctagatttgtgaaagatggcaaagta | | Inserting *cat* into Janus | |  |
| Chloramphenicol_For_HindIII | ggaagcttagtgggatatttttaaaatat | | Inserting *cat* into Janus | |  |
| Chloramphenicol_Rev_Xbal | ggtctagattataaaagccagtcattagg | | Inserting *cat* into Janus | |  |
| Chloramphenicol_Rev_EcoRV | aagatatcttataaaagccagtcattagg | | Inserting *cat* into Janus | |  |
| trdii_inf_KO_F_BamHI | aaggatccgcaatccaaaaatctctgga | | Removing P1 repeat | |  |
| trdii_inf_KO_F_ApaL | aagggcccgcaatccaaaaatctctgga | | Removing P1 repeat | |  |
| trdii_inf_up_R_ApaI | aagggcccatatttctgattttctaatg | | Removing P1 repeat | |  |
| XerC/S_Up_KO_F | gcgaaggagaagttacacttatcgaa | | Removing *xerS* | |  |
| XerC/S_Up_KO_ApaL_R | aagggcccgtattttctccataagtaagt | | Removing *xerS* | |  |
| XerC/S_Down_KO_BamHI_F | aaggatcc**actgacctctatacccatatt** | | Removing *xerS* | |  |
| XerC/S_Down_KO_R | ttcgccgtattagtggtggtggagct | | Removing *xerS* | |  |
| xerD_KO_UP_F | tcagtcaaccacgaggtaccatcaga | | Removing *xerD* | |  |
| xerD_KO_UP_Apal_R | aagggccccttataggactgcttggaatt | | Removing *xerD* | |  |
| xerD_KO_DOWN_BamHI_F | aaggatcctcagctcaagtcttacgtgaa | | Removing *xerD* | |  |
| xerD_KO_DOWN_R | tctatcaagtctagctttgaggcact | | Removing *xerD* | |  |
| SS_Integrase_KO_UP_F | cactgctacttatgcagtgggaatt | | Removing *ccrB* | |  |
| SS_Integrase_KO_UP_R_ApaI | aagggcccttgtgcctctaacgaatacc | | Removing *ccrB* | |  |
| SS_Integrase_KO_Down_F_BamHI | aaggatccggacaatggctaaaatccat | | Removing *ccrB* | |  |
| SS_Integrase_KO_Down_R | gtgacacaagaaatgccaaacttcg | | Removing *ccrB* | |  |
| RexA/B_UP_KO_F | ctttaaccagccataaatggtattacgtg | | Removing *rexAB* | |  |
| RexA/B_UP_KO_R_Apal | aagggcccgttgggggcaatgtagaagac | | Removing *rexAB* | |  |
| RexA/B_DOWN_KO_F_BamHI | aaggatcctggctatctgctttacgaaaac | | Removing *rexAB* | |  |
| RexA/B_DOWN_KO_R | gaagtcgctcgtcaaagtcactttta | | Removing *rexAB* | |  |
| RecG_KO_Up_F | gattgattcagggatgggacggattgg | | Removing *recG* | |  |
| RecG_KO_Up_R_ApaL | aagggcccgctccactcctttcttttcta | | Removing *recG* | |  |
| RecG_KO_Down_F_BamHI | aaggatccgctttctctaaggaaaacttaag | | Removing *recG* | |  |
| RecG_KO_Down_R | gctagaaagacgcttctcatcaacctga | | Removing *recG* | |  |
| recU_KO_UP_F | cttcaacttgattgatgacttccttg | | Removing *recU* | |  |
| recU_KO_UP_R_ApaL | aagggcccacaaaccaacgattctgcgcc | | Removing *recU* | |  |
| recU_KO_Down_F_EcorV | aagatatcaattctccttattggtacaat | | Removing *recU* | |  |
| recU_KO_Down_R | ggcgtttcaaaatatcaaaattcgtc | | Removing *recU* | |  |
| recA_KO_UP_F | gacggagtgacctatgtcgtccttcc | | Removing *recA* | |  |
| recA_KO_UP_R_ApaI | aagggccctctattctcctacattctaat | | Removing *recA* | |  |
| recA_KO_Down_F_BamHI | aaggatccgaagaagcagtgaatgaagaa | | Removing *recA* | |  |
| recA_KO_Down_R | gcagagaaccgctgataaaccttgcg | | Removing *recA* | |  |
| DprA _KO_up_L | gaataggtgtcatcaaaggagagattaac | | Removing *dprA* | |  |
| DprA_KO_up_ApaI_R | ttgggccctgaacccaacatttccataat | | Removing *dprA* | |  |
| DprA_KO_down_BamHI_L | ttggatccactccatttctttttctactc | | Removing *dprA* | |  |
| DprA_KO_down_R | cctatcttgtgattgtgctcttctctc | | Removing *dprA* | |  |
| clpP_UP_F | aattgaagttattaatcacccactga | | Removing *clpP* | |  |
| clpP_UP_R_ApaL | aagggcccagaacgttctccacggcttgt | | Removing *clpP* | |  |
| clpP_Down_F_BamHI | aaggatccagcgcccaggaaacacttgaa | | Removing *clpP* | |  |
| clpP_Down_R  hexB_KO_Up_F  hexB_KO_Up_R_ApaI  hexB_KO_Down_F-BamHI  hexB_KO_Down_R  Tet_F_ApaI_Ii6_IViii6  Tet_R_BamHI_Ii6_IViii6  hsdR_KO_UP_R_Apal_V2  hsdR_KO_DOWN_F  hsdR_KO_Down_R  antitoxin_IF_KO_Up_R_ApaI  antitoxin_IF_KO_Down_F_BamHI | aatggaacacctgcttttgtagcgttc  ggcagctgcatcgtgaaatacttgtt  aagggcccaataatatgagacatctttca  aaggatccttggggaaatattaaaagtat  atgggtcacattttaaatatggagga  aagggcccgataaccactatcgataaccactatctcattggtactcctaatcacgtactctctttgat  aaggatccgacggttgttatcgacggttgttatctcaccaaatctccctaagttattttattgaacat  aagggcccttttttctcactttaaacaaatagtttagct  aggatccgagacgaaagtgttaacgcaa  atgtctgtcttatcatctgtgaag  aagggcccttttactaccatctgatttcc  aaggatccaaagctttggtggaaaaatga | | Removing *clpP*  Removing *hexB*  Removing *hexB*  Removing *hexB*  Removing *hexB*  Construction of Mega::*tetM*  Construction of Mega::*tetM*  Removing *hsdR*  Removing *hsdR*  Removing *hsdR*  Removing antitoxin  Removing antitoxin | |  |
|  | |  |  |  |  |
|  | |  |  |  |  |
|  |  | |  |  | |
|  |  | |  |  | |
|  |  | |  |  | |
|  |  | |  |  | |

|  |  |  |  |
| --- | --- | --- | --- |

**Supplementary Table S3** Modified motifs identified with single molecule real-time sequencing data. The modified motifs in strains characterized through SMRT sequencing with a mean score above 30. Strains are named according to their genotype; for Δ*tvrR* mutants, this includes a designation of whether they were the dominant, or rare, arrangement in culture.

| **Strain** | **Accession code** | **Restriction modification system** | **RMS Type** | **SpnIV HsdS TRDs** | **Motif** | **Motif group** | **Modified base** | **No. of modified motifs** | **No. of motifs in genome** | **Mean score of motifs** |
| --- | --- | --- | --- | --- | --- | --- | --- | --- | --- | --- |
| RMV1 *rpsL** Δ*tvrR* | ERR2576015 | SpnIII | I | - | CACNNNNNNNCTK | CACNNNNNNNCTK/MAGNNNNNNNGTG | m6A | 979 | 1264 | 74.15 |
|  |  | SpnIII | I | - | MAGNNNNNNNGTG | CACNNNNNNNCTK/MAGNNNNNNNGTG | m6A | 968 | 1264 | 76.14 |
|  |  | Orphan MTase | Unknown | - | CTBVAG | CTBVAG | m6A | 4846 | 9054 | 124.08 |
|  |  | SpnV | II | - | DTCGAG | DTCGAG | m6A | 1301 | 1337 | 139.74 |
|  |  | SpnD39I | II | - | TCTAGA | TCTAGA | m6A | 587 | 622 | 124.95 |
| RMV1 *rpsL** *tvr:*:*cat* | ERR2831552 | Orphan MTase | Unknown | - | CTBVAG | CTBVAG | m6A | 4981 | 9054 | 85.92 |
|  |  | SpnV | II | - | DTCGAG | DTCGAG | m6A | 1306 | 1337 | 94.33 |
|  |  | SpnIII | I | - | GAANNNNNNNNNTTYG | GAANNNNNNNNNTTYG/CRAANNNNNNNNNTTC | m6A | 942 | 995 | 71.35 |
|  |  | SpnIII | I |  | CRAANNNNNNNNNTTC | GAANNNNNNNNNTTYG/CRAANNNNNNNNNTTC | m6A | 930 | 995 | 69.07 |
|  |  | SpnD39I |  |  | TCTAGANNW | TCTAGANNW | m6A | 403 | 409 | 95.67 |
|  |  | SpnD39I |  |  | TCTAGANAS | TCTAGANAS | m6A | 70 | 71 | 100.41 |
| RMV10 *rpsL** | ERR2576023 | SpnIII | I | - | CAGNNNNNNNGTG | CACNNNNNNNCTG/CAGNNNNNNNGTG | m6A | 417 | 446 | 118.13 |
|  |  | SpnIII | I | - | CACNNNNNNNCTG | CACNNNNNNNCTG/CAGNNNNNNNGTG | m6A | 421 | 446 | 109.26 |
|  |  | SpnIII | I | - | CACNNNNNNNNTTC | CACNNNNNNNNTTC/GAANNNNNNNNGTG | Unknown | 139 | 643 | 40.88 |
|  |  | SpnIII | I | - | GAANNNNNNNNGTG | CACNNNNNNNNTTC/GAANNNNNNNNGTG | Unknown | 109 | 643 | 42.65 |
|  |  | Unknown | Unknown | - | CNNAAANNCGNGTNNA | CNNAAANNCGNGTNNA | m4C | 5 | 6 | 42.80 |
|  |  | SpnVI | II | - | GATGC | GCATC/GATGC | m6A | 3139 | 3235 | 163.34 |
|  |  | SpnVI | II | - | GCATC | GCATC/GATGC | m6A | 3150 | 3235 | 161.70 |
|  |  | Diguanosine modification | Unknown | - | GG | GG | Unknown | 2078 | 172947 | 39.22 |
|  |  | SpnD39I | II | - | TCTAGA | TCTAGA | m6A | 639 | 668 | 133.18 |
| RMV11 rpsL* | ERR2576020 | SpnIII | I | - | CAGNNNNNNNNTTYG | CAGNNNNNNNNTTYG | Unknown | 191 | 732 | 44.48 |
|  |  | SpnIII | I | - | AAGNNNNNNNNTTYG | CRAANNNNNNNNCTT/AAGNNNNNNNNTTYG | m6A | 993 | 1021 | 148.42 |
|  |  | SpnIII | I | - | CRAANNNNNNNNCTT | CRAANNNNNNNNCTT/AAGNNNNNNNNTTYG | m6A | 999 | 1021 | 136.31 |
|  |  | SpnVI | II | - | GATGC | GATGC/GCATC | m6A | 3188 | 3209 | 202.94 |
|  |  | SpnVI | II | - | GCATC | GATGC/GCATC | m6A | 3182 | 3209 | 200.22 |
|  |  | SpnVII | II | - | GGCCBBVNB | GGCCBBVNB | m4C | 172 | 1023 | 51.62 |
|  |  | SpnVII | II | - | GGCCMNB | GGCCMNB | Unknown | 374 | 1836 | 45.02 |
|  |  | SpnD39I | II | - | TCTAGA | TCTAGA | m6A | 356 | 652 | 61.74 |
| RMV3 *rpsL** Δ*tvrR* | ERR2576026 | SpnIII | I | - | CACNNNNNNNDTTC | CACNNNNNNNDTTC/GAAHNNNNNNNGTG | m6A | 394 | 509 | 51.37 |
|  |  | SpnIII | I | - | GAAHNNNNNNNGTG | CACNNNNNNNDTTC/GAAHNNNNNNNGTG | m6A | 314 | 509 | 46.74 |
|  |  | SpnIV | I | I,iii | GAYNNNNNNTCC | GAYNNNNNNTCC/GGANNNNNNRTC | m6A | 1600 | 1612 | 245.27 |
|  |  | SpnIV | I | I,iii | GGANNNNNNRTC | GAYNNNNNNTCC/GGANNNNNNRTC | m6A | 1596 | 1612 | 239.86 |
|  |  | SpnIII | I | - | MAGNNNNNNNGTG | MAGNNNNNNNGTG/CACNNNNNNNCTK | m6A | 989 | 1242 | 108.49 |
|  |  | SpnIII | I | - | CACNNNNNNNCTK | MAGNNNNNNNGTG/CACNNNNNNNCTK | m6A | 971 | 1242 | 106.09 |
|  |  | SpnV | II | - | TCGAG | TCGAG | m6A | 1548 | 1556 | 239.47 |
|  |  | SpnD39I | II | - | TCTAGA | TCTAGA | m6A | 683 | 692 | 237.78 |
| RMV4 *rpsL** Δ*tvrR* | ERR2576029 | SpnIII | I | - | CACNNNNNNNNTTC | CACNNNNNNNNTTC/GAANNNNNNNNGTG | m6A | 660 | 665 | 255.68 |
|  |  | SpnIII | I | - | GAANNNNNNNNGTG | CACNNNNNNNNTTC/GAANNNNNNNNGTG | m6A | 659 | 665 | 236.07 |
|  |  | SpnIV | I | I,i | GAYNNNNNNTATC | GATANNNNNNRTC/GAYNNNNNNTATC | m6A | 757 | 759 | 289.94 |
|  |  | SpnIV | I | I,i | GATANNNNNNRTC | GATANNNNNNRTC/GAYNNNNNNTATC | m6A | 758 | 759 | 286.30 |
|  |  | SpnIII | I | - | HCACNNNNNNNCTG | HCACNNNNNNNCTG | Unknown | 110 | 361 | 41.40 |
|  |  | SpnD39I | II | - | TCTAGA | TCTAGA | m6A | 709 | 714 | 263.87 |
| RMV5 *rpsL** Δ*tvrR* (dominant) | ERR2576021 | SpnIII | I | - | AAGNNNNNNNNTTYG | AAGNNNNNNNNTTYG/CRAANNNNNNNNCTT | m6A | 779 | 1042 | 64.79 |
|  |  | SpnIII | I | - | CRAANNNNNNNNCTT | AAGNNNNNNNNTTYG/CRAANNNNNNNNCTT | m6A | 729 | 1042 | 61.63 |
|  |  | Unknown | Unknown | - | BNNNNNNNNTTTTATAC | BNNNNNNNNTTTTATAC | Unknown | 27 | 89 | 43.70 |
|  |  | SpnIII | I | - | CAGNNNNNNNGTG | CACNNNNNNNCTG/CAGNNNNNNNGTG | m6A | 406 | 455 | 90.80 |
|  |  | SpnIII | I | - | CACNNNNNNNCTG | CACNNNNNNNCTG/CAGNNNNNNNGTG | m6A | 408 | 455 | 82.56 |
|  |  | SpnIII | I | - | CACNNNNNNNCTTNH | CACNNNNNNNCTTNH | Unknown | 118 | 693 | 40.45 |
|  |  | SpnIII | I | - | CAGNNNNNNNNTTYG | CAGNNNNNNNNTTYG | Unknown | 200 | 736 | 42.17 |
|  |  | Unknown | Unknown | - | CGNTGTAVNA | CGNTGTAVNA | Unknown | 23 | 91 | 52.17 |
|  |  | Unknown | Unknown | - | DNAAGNNBNNNNGTG | DNAAGNNBNNNNGTG | Unknown | 102 | 462 | 41.75 |
|  |  | SpnIV | I | IV,iii | TGANNNNNNNTATC | GATANNNNNNNTCA/TGANNNNNNNTATC | m6A | 608 | 640 | 183.86 |
|  |  | SpnIV | I | IV,iii | GATANNNNNNNTCA | GATANNNNNNNTCA/TGANNNNNNNTATC | m6A | 610 | 640 | 182.12 |
|  |  | SpnD39I | II | - | TCTAGA | TCTAGA | m6A | 608 | 654 | 174.17 |
| RMV5 *rpsL** Δ*tvrR* (rare) | ERR2576028 | SpnIII | I | - | CAGNNNNNNNGTG | CAGNNNNNNNGTG/CACNNNNNNNCTG | m6A | 435 | 436 | 257.74 |
|  |  | SpnIII | I | - | CACNNNNNNNCTG | CAGNNNNNNNGTG/CACNNNNNNNCTG | m6A | 435 | 436 | 245.60 |
|  |  | Diguanosine modification | Unknown | - | DNNNNNCGGTGTVNND | DNNNNNCGGTGTVNND | Unknown | 47 | 168 | 54.77 |
|  |  | SpnIV | I | I,i | GAYNNNNNNTATC | GATANNNNNNRTC/GAYNNNNNNTATC | m6A | 753 | 753 | 275.80 |
|  |  | SpnIV | I | I,i | GATANNNNNNRTC | GATANNNNNNRTC/GAYNNNNNNTATC | m6A | 753 | 753 | 270.57 |
|  |  | SpnD39I | II | - | TCTAGA | TCTAGA | m6A | 700 | 704 | 263.52 |
| RMV6 *rpsL** Δ*tvrR* (dominant) | ERR2576016 | Unknown | Unknown | - | ANCGNTNTAGNANNA | ANCGNTNTAGNANNA | Unknown | 5 | 6 | 51.20 |
|  |  | SpnIII | I | - | CACNNNNNNNNTTC | CACNNNNNNNNTTC/GAANNNNNNNNGTG | m6A | 647 | 687 | 108.90 |
|  |  | SpnIII | I | - | GAANNNNNNNNGTG | CACNNNNNNNNTTC/GAANNNNNNNNGTG | m6A | 629 | 687 | 103.25 |
|  |  | SpnIII | I | - | CAGNNNNNNNGTG | CAGNNNNNNNGTG/CACNNNNNNNCTG | Unknown | 252 | 447 | 48.84 |
|  |  | SpnIII | I | - | CACNNNNNNNCTG | CAGNNNNNNNGTG/CACNNNNNNNCTG | Unknown | 191 | 447 | 46.42 |
|  |  | Diguanosine modification | Unknown | - | CGGTGTA | CGGTGTA | Unknown | 19 | 86 | 46.58 |
|  |  | Unknown | Unknown | - | CTCNCGTNNNNANTNT | CTCNCGTNNNNANTNT | Unknown | 15 | 17 | 63.67 |
|  |  | SpnIV | I | II, iii | TCANNNNNNNTCC | GGANNNNNNNTGA/TCANNNNNNNTCC | m6A | 1080 | 1110 | 163.33 |
|  |  | SpnIV | I | II, iii | GGANNNNNNNTGA | GGANNNNNNNTGA/TCANNNNNNNTCC | m6A | 1083 | 1110 | 156.51 |
|  |  | SpnD39I | II | - | TCTAGA | TCTAGA | m6A | 627 | 650 | 139.05 |
| RMV6 *rpsL** Δ*tvrR* (rare) | ERR2576017 | SpnIII | I | - | CAGNNNNNNNGTG | CAGNNNNNNNGTG/CACNNNNNNNCTG | m6A | 435 | 436 | 163.49 |
|  |  | SpnIII | I | - | CACNNNNNNNCTG | CAGNNNNNNNGTG/CACNNNNNNNCTG | m6A | 435 | 436 | 156.29 |
|  |  | Diguanosine modification | Unknown | - | GG | GG | Unknown | 3711 | 172981 | 39.45 |
|  |  | SpnIV | I | III,i | TCANNNNNNNTCC | GGANNNNNNNTGA/TCANNNNNNNTCC | m6A | 1094 | 1094 | 181.59 |
|  |  | SpnIV | I | III,i | GGANNNNNNNTGA | GGANNNNNNNTGA/TCANNNNNNNTCC | m6A | 1094 | 1094 | 175.30 |
|  |  | SpnD39I | II | - | TCTAGA | TCTAGA | m6A | 700 | 704 | 168.47 |
| RMV7 *rpsL** *tvrR*::Janus (dominant) | ERR2576030 | SpnIII | I | - | AAGNNNNNNNNTTYG | AAGNNNNNNNNTTYG/CRAANNNNNNNNCTT | m6A | 1010 | 1020 | 107.71 |
|  |  | SpnIII | I | - | CRAANNNNNNNNCTT | AAGNNNNNNNNTTYG/CRAANNNNNNNNCTT | m6A | 1005 | 1020 | 100.18 |
|  |  | SpnIII | I | - | CAGNNNNNNNNTTYG | CAGNNNNNNNNTTYG | Unknown | 202 | 728 | 40.34 |
|  |  | SpnIV | I | III,iii | TGANNNNNNNTCC | GGANNNNNNNTCA/TGANNNNNNNTCC | m6A | 1023 | 1024 | 156.03 |
|  |  | SpnIV | I | III,iii | GGANNNNNNNTCA | GGANNNNNNNTCA/TGANNNNNNNTCC | m6A | 1023 | 1024 | 152.69 |
|  |  | SpnD39I | II | - | TCTAGA | TCTAGA | m6A | 650 | 650 | 149.26 |
| RMV7 *rpsL** Δ*tvrR* (rare) | ERR2576027 | SpnIII | I | - | CACNNNNNNNNTTC | CACNNNNNNNNTTC/GAANNNNNNNNGTG | Unknown | 361 | 672 | 46.64 |
|  |  | SpnIII | I | - | GAANNNNNNNNGTG | CACNNNNNNNNTTC/GAANNNNNNNNGTG | Unknown | 285 | 672 | 45.63 |
|  |  | SpnIII | I | - | CAGNNNNNNNGTG | CAGNNNNNNNGTG/CACNNNNNNNCTG | m6A | 442 | 445 | 104.11 |
|  |  | SpnIII | I | - | CACNNNNNNNCTG | CAGNNNNNNNGTG/CACNNNNNNNCTG | m6A | 440 | 445 | 95.07 |
|  |  | SpnIII | I | - | CAGNNNNNNNNTTYG | CAGNNNNNNNNTTYG/CRAANNNNNNNNCTG | m6A | 686 | 724 | 71.67 |
|  |  | SpnIII | I | - | CRAANNNNNNNNCTG | CAGNNNNNNNNTTYG/CRAANNNNNNNNCTG | m6A | 607 | 724 | 56.82 |
|  |  | Unknown | Unknown | - | CGNNNNNAANATGAC | CGNNNNNAANATGAC | Unknown | 14 | 16 | 87.86 |
|  |  | SpnIV | I | III,i | TGANNNNNNNTATC | GATANNNNNNNTCA/TGANNNNNNNTATC | m6A | 617 | 617 | 210.69 |
|  |  | SpnIV | I | III,i | GATANNNNNNNTCA | GATANNNNNNNTCA/TGANNNNNNNTATC | m6A | 617 | 617 | 209.99 |
|  |  | Unknown | Unknown | - | GTTNNANNNATGNCG | GTTNNANNNATGNCG | Unknown | 13 | 15 | 58.00 |
|  |  | SpnD39I | II | - | TCTAGA | TCTAGA | m6A | 655 | 656 | 204.15 |
| RMV8 *rpsL** Δ*tvrR* (dominant) | ERR2576019 | SpnIII | I | - | CAGNNNNNNNGTG | CAGNNNNNNNGTG/CACNNNNNNNCTG | m6A | 401 | 474 | 56.05 |
|  |  | SpnIII | I | - | CACNNNNNNNCTG | CAGNNNNNNNGTG/CACNNNNNNNCTG | m6A | 385 | 474 | 55.14 |
|  |  | SpnIV | I | IV,ii | TCANNNNNNRTAC | TCANNNNNNRTAC/GTAYNNNNNNTGA | m6A | 525 | 601 | 56.54 |
|  |  | SpnIV | I | IV,ii | GTAYNNNNNNTGA | TCANNNNNNRTAC/GTAYNNNNNNTGA | m6A | 511 | 601 | 56.22 |
|  |  | SpnD39I | II | - | TCTAGA | TCTAGA | m6A | 623 | 732 | 55.62 |
| RMV8 *rpsL** Δ*tvrR* (rare) | ERR2576024 | SpnIII | I | - | CAGNNNNNNNNTTYG | CAGNNNNNNNNTTYG/CRAANNNNNNNNCTG | m6A | 742 | 742 | 178.22 |
|  |  | SpnIII | I | - | CRAANNNNNNNNCTG | CAGNNNNNNNNTTYG/CRAANNNNNNNNCTG | m6A | 741 | 742 | 143.29 |
|  |  | Unknown | Unknown | - | CGNTANNANTANNANG | CGNTANNANTANNANG | Unknown | 7 | 9 | 63.14 |
|  |  | SpnIV | I | I,i | GAYNNNNNNTATC | GATANNNNNNRTC/GAYNNNNNNTATC | m6A | 797 | 797 | 185.62 |
|  |  | SpnIV | I | I,i | GATANNNNNNRTC | GATANNNNNNRTC/GAYNNNNNNTATC | m6A | 797 | 797 | 181.80 |
|  |  | SpnD39I | II | - | TCTAGA | TCTAGA | m6A | 730 | 730 | 179.08 |
|  |  | Unknown | Unknown | - | TTTATACYW | TTTATACYW | Unknown | 36 | 128 | 42.17 |
| RMV9 *rpsL** Δ*tvrR* (rare) | ERR2576025 | SpnIII | I | - | AAGNNNNNNNNTTYG | AAGNNNNNNNNTTYG/CRAANNNNNNNNCTT | m6A | 1089 | 1093 | 160.32 |
|  |  | SpnIII | I | - | CRAANNNNNNNNCTT | AAGNNNNNNNNTTYG/CRAANNNNNNNNCTT | m6A | 1089 | 1093 | 148.14 |
|  |  | Unknown | Unknown | - | CGNTGTANNANNNNH | CGNTGTANNANNNNH | Unknown | 22 | 92 | 54.77 |
|  |  | Diguanosine modification | Unknown | - | DCGGTGTNNNDNNNNNA | DCGGTGTNNNDNNNNNA | Unknown | 21 | 97 | 38.57 |
|  |  | SpnD39I | II | - | TCTAGA | TCTAGA | m6A | 746 | 746 | 182.99 |

**Supplementary Table S4** Representative protein sequences of TRDs.

| **TRD label** | **TRD type** | **Representative protein sequence** |
| --- | --- | --- |
| I | N-terminal | KKVKLGEVLSLKKGKKATVLAEQTTLSQRYIQIDDLRNNNNLKFTESLNMTEALPDDILIAWDGANAGTVGYGLSGAVGSTITVLKKNERYKEKIISDYLGVFLESKSQYLREHSTGATIPHLNKNILLDLQLELLGIEEQENIICILNTIKGLITKRKLQ |
| II | N-terminal | KKVKLGQVATFINGYAFKPQDWSSEGKEIIRIQNLTKTSKGINYYSGTIDKKYIVEAGDILISWSGTLGVFQWCGRSAVLNQHIFKVVFDKIDIDKSYFKYVVEKGLQDAVKHTHGSTMKHLTKKYFDNIIVPYTNLGEQQRIASELDLLSKLILRRQEQLEEL |
| III | N-terminal | KKVKLGEVCEILSGYAFKSSQFNDNKIGLPLIRIRDVERGFSDTYFEGTYPEEYLIKNGDLLITMDGSFILKKWEGDLALLNQRVCKIKITDKSVDEGYISWLIPKFLKEIEDKTPFVTVKHLSVAKIKDISFVLPNKLEQKLIAKKLNTISQ |
| IV | N-terminal | KKVKLGEVATFINGYAFKPQDWSSEGKEIIRIQNLTKTSTEINYYSGTIDKKYIVEAGDILISWSGTLGVFQWRGRSAVLNQHIFKVVFDKIDIDKSYFKYVVEKGLQDAVKHTHGSTMKHLTKKYFDNIIVPYTNLGEQQRIASELDLLSKLILRRQEQLEEL |
| i | C-terminal | NLFDIIDGDRGKNYPKSDELFSEEYCLFLNTKNVTKNGFSFDTKQFITKTKDKLLRKGKLERYDIVLTTRGTVGNVAYYDELIKYKHLRINSGMVILRPKTPNLNQKFIIHVLRNNNYSRVISGSAQPQLPITKLKKILLPLPPLALQNEFADFVAQVDKSQLAIQKSLEE |
| ii | C-terminal | KTGQQCFKFSSGKFLDKHDRVFEGYPAYGGNGIAWKSRKYLIDNPTIIIGRVGAYCGNVRTTHGKVWISDNAIYIKEFKNSDFNLVFLLELMKVIDFSKFADFSGQPKITQKPLENQKYILPPLALKNEFADFVALVDKSQLAIQKSLEE |
| iii | C-terminal | EKEWKVSKWNEILTIRNGKNQKQVEDADGKFPIYGSGGIMGYAKDWIVKKNSVIIGRKGNINKPILVRENFWNVDTAFGLEPVLEKINSEYLFYFCQLYNFEKLNKAVTIPSLTKSDLLNISIPLPPLALQNEFADFVVQVDKSQLAIQKSLEE |
| iv | C-terminal | KTGQQCFKFSSGKFLDKHDRVFEGYPAYGGNGIAWKSRKYLIDNPTITIG |
